# Global cooling induced by biophysical effects of bioenergy crop cultivation

**DOI:** 10.1038/s41467-021-27520-0

**Published:** 2021-12-13

**Authors:** Jingmeng Wang, Wei Li, Philippe Ciais, Laurent Z. X. Li, Jinfeng Chang, Daniel Goll, Thomas Gasser, Xiaomeng Huang, Narayanappa Devaraju, Olivier Boucher

**Affiliations:** 1 Department of Earth System Science, Ministry of Education Key Laboratory for Earth System Modeling, Institute for Global Change Studies, Tsinghua University, Beijing, 100084 China; 2 Institute for Carbon Neutrality, Tsinghua University, Beijing, 100084 China; 3 Laboratoire des Sciences du Climat et de l’Environnement, LSCE/IPSL, CEA-CNRS-UVSQ, Université Paris-Saclay, 91191 Gif-sur-Yvette, France; 4 Laboratoire de Météorologie Dynamique, Centre National de la Recherche Scientifique, Sorbonne Université, Ecole Normale Supérieure, Ecole Polytechnique, 75252 Paris, France; 5 College of Environmental and Resource Sciences, Zhejiang University, Hangzhou, China; 6 Université Paris Saclay, CEA-CNRS-UVSQ, LSCE/IPSL, Gif sur Yvette, France; 7 International Institute for Applied Systems Analysis (IIASA), Laxenburg, Austria; 8 Institut Pierre-Simon Laplace, Sorbonne Université/IPSL, Paris, France

**Keywords:** Climate change, Climate-change mitigation

## Abstract

Bioenergy crop with carbon capture and storage (BECCS) is a key negative emission technology to meet carbon neutrality. However, the biophysical effects of widespread bioenergy crop cultivation on temperature remain unclear. Here, using a coupled atmosphere-land model with an explicit representation of lignocellulosic bioenergy crops, we find that after 50 years of large-scale bioenergy crop cultivation following plausible scenarios, global air temperature decreases by 0.03~0.08 °C, with strong regional contrasts and interannual variability. Over the cultivated regions, woody crops induce stronger cooling effects than herbaceous crops due to larger evapotranspiration rates and smaller aerodynamic resistance. At the continental scale, air temperature changes are not linearly proportional to the cultivation area. Sensitivity tests show that the temperature change is robust for eucalypt but more uncertain for switchgrass among different cultivation maps. Our study calls for new metrics to take the biophysical effects into account when assessing the climate mitigation capacity of BECCS.

## Abstract (web summary)

Bioenergy crops has been proposed as a climate mitigation measure, but how the biophysical effects of large-scale cultivation would influence the climate is not well known. Here, the authors use models to show that large-scale cultivation could cool the global land by 0.03 to 0.08 °C.

## Introduction

Large-scale bioenergy crop cultivation with carbon capture and storage (BECCS) has been identified by integrated assessment models (IAMs) as a major negative emission technology (NET) for removing CO_2_ from the atmosphere^1–3^. Most mitigation scenarios which limit the global temperature increase below 2 or 1.5 °C in 2100 assume widespread deployment of this NET^2,4^. In the scenarios where BECCS was the only land-based negative emission option^5,6^, about 128 PgC needs to be removed through BECCS to reach the temperature limiting target under Representative Concentration Pathway 2.6 (RCP 2.6). However, the real mitigation potential of BECCS depends on multiple dimensions, such as achievable bioenergy crop yields^7^, available cultivation lands^8^, land use change emissions^5,6^, and supply of water^9,10^ and nutrients^11–13^. Marginal lands (e.g., abandoned agricultural land or degraded lands) are targeted for bioenergy crop cultivation to avoid land competition with food crops and forests^8,14,15^, but large-scale use of current agricultural systems for bioenergy crops was also proposed^12,16,17^. In addition, the biophysical impacts of bioenergy crops are important to the mitigation potential of BECCS. Biophysically cooling effects were found in the US when converting annual crops to perennial bioenergy crops^18^, but the biophysical interactions between large-scale bioenergy crop cultivation and climate remain unclear at the global scale. Biophysical climate effects could either partly offset or enhance the climate benefits from carbon-dioxide removal (CDR). Changes in land management and land cover alter land surface properties like albedo and aerodynamic resistance and influence the surface energy budget^19–23^. In turn, these changes modify air temperature (T_a_) locally and regionally, in the latter case through atmospheric circulation changes and teleconnection mechanisms^24^. It is thus important to quantify the biophysical climate effects of large-scale bioenergy crops to fully assess their role in climate mitigation.

Here, we simulated the biophysical climate impact of a range of future bioenergy crop cultivation scenarios (Supplementary Methods 1) using an Earth system model (IPSL-CM^25^). IPSL-CM deploys a land surface scheme with an explicit representation of the main lignocellulosic bioenergy crops (ORCHIDEE-MICT-BIOENERGY^26^) as four plant functional types (PFTs): eucalypt, poplar & willow (sharing the same PFT because of their similar ecological processes and parameters), miscanthus, and switchgrass^26^. The resolved management practices include the periodic harvest of woody crops, separate harvested biomass pools, and regrowth from belowground biomass after harvest. The parameters controlling physiological processes, growth, and phenology were calibrated using plant measurements and a global database of yields from field trials^26,27^. Model performance was evaluated against those field measured yields^27^ and observation-based biophysical variables, such as albedo and evapotranspiration, which control the energy budget (see Supplementary Methods 2, Supplementary Tables 1 and 2, and Supplementary Figs. 1–9). From the coupled simulations, we find that large-scale bioenergy crop cultivation induces a biophysical cooling effect at the global scale, but the air temperature change (ΔT_a_) has strong spatial variations and interannual variability. Compared to the herbaceous crops, changes in the energy fluxes induced by woody crops in the cultivation regions are larger, and the cooling effect is more robust across different cultivation maps.

## Results

### Spatial patterns of biophysical ΔT_a_

**Fig. 1:**
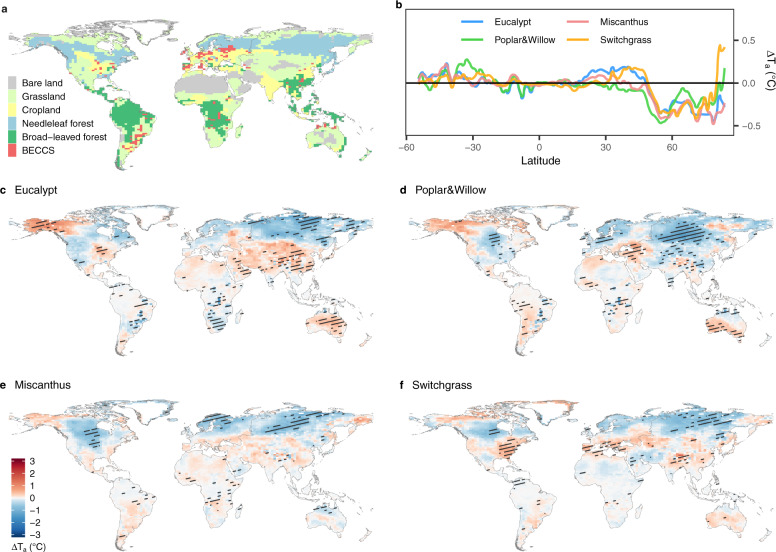
Bioenergy crop cultivation map and air temperature change over land. **a** The composite bioenergy crop cultivation map where red grid cells represent the bioenergy crop cultivation regions (i.e., BECCS regions) and the main vegetation type in each grid cell is shown outside the BECCS regions. **c**–**f** Air temperature changes (ΔT_a_) induced by cultivation of each bioenergy crop type based on the composite cultivation map with their zonal averages shown in (**b**). Black shading in **c**–**f** indicates grid cells with significant (*p* < 0.1) change in air temperature.

A composite map (Supplementary Fig. 10f) for bioenergy crop deployment around the world was generated from three marginal land datasets^8,14^ and two bioenergy cultivation maps from two different IAMs^17^ (see Supplementary Fig. 10a–e and Supplementary Methods 3). The global total bioenergy crop cultivation area in the composite cultivation map is 466 M ha, distributed between 38°S to 60°N (the BECCS regions in Fig. 1a). Based on the composite map, we performed five 50-year simulations with the IPSL-CM model to simulate the biophysical effects of different bioenergy crop cultivation scenarios (more details in Supplementary Methods 4.1 and Supplementary Figs. 10–18): a reference scenario (S_ref_) where grid cells in the BECCS regions are fully occupied by food crops (because current marginal land areas are low yield croplands, or recently abandoned arable land, see Supplementary Methods 3 and Supplementary Fig. 11), and four idealized bioenergy crop scenarios (i.e., S_euc_, S_p&w_, S_mis_, and S_swi_) where all BECCS grid cells are assumed to be fully covered by a single type of the four bioenergy crops (i.e., eucalypt, poplar & willow, miscanthus, and switchgrass), with the remaining land cover being the same as in S_ref_. Differences (∆) in simulated climate between each bioenergy scenario and the reference scenario represent the biophysical climate effects of each bioenergy crop (see “Methods” and Supplementary Methods 4.1).

**Fig. 2:**
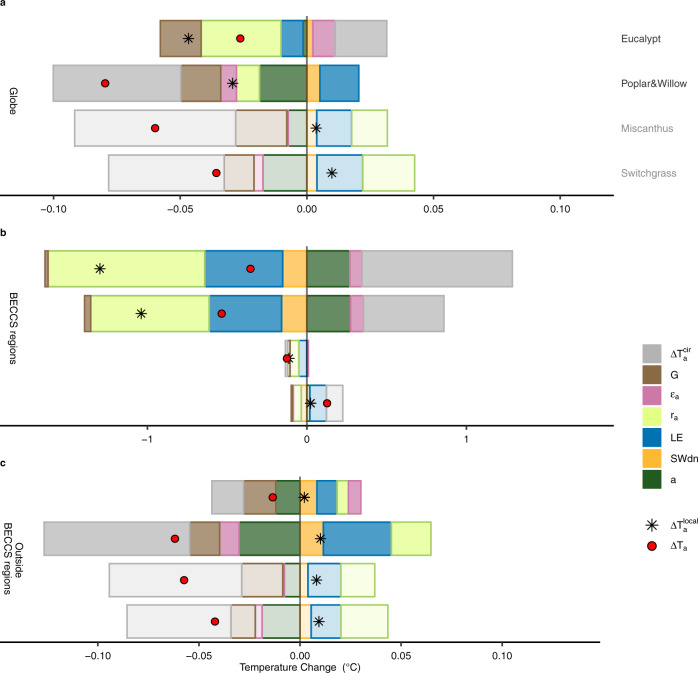
Contributions of different components to air temperature changes. Three panels represent the results at the global scale (**a**), in the BECCS regions (**b**), and outside the BECCS regions (**c**). In each panel, four groups of bars represent four idealized bioenergy crop scenarios (i.e., eucalypt, poplar & willow, miscanthus, and switchgrass) based on the composite cultivation map. Bars for $\Delta {{{{{{\rm{T}}}}}}}_{{{{{{\rm{a}}}}}}}^{{{{{{\rm{cir}}}}}}}$, G, ε_a_, r_a_, LE, SW_dn_, and α represent different components that contribute to the air temperature change, i.e., the altered atmospheric circulation, ground heat flux, longwave radiative emissivity of air, aerodynamic resistance, latent heat flux, surface downward shortwave radiation, and surface albedo, respectively. The net air temperature change is shown by red dots, which is the sum of all bars. Black asterisks represent the contribution of altered local surface energy balance, which is the sum of the colored bars excluding the gray bar. Note that the scale of the x-axis is different for the three panels.

Although the assumed bioenergy crop cultivation accounts for 3.8% of global land area in the composite cultivation map (Fig. 1a), air temperature is significantly (*p* < 0.1) changed over about 10% of the global land area (grid cells with black shading in Fig. 1c–f, 84–89% of which are distributed outside of the BECCS regions). At the global scale, different bioenergy crop cultivations result in cooling effects with ΔT_a_ = −0.03, −0.08, −0.06, and −0.04 °C for S_euc_, S_p&w_, S_mis_, and S_swi_, respectively (Figs. 1c–f and 2a), when averaged over the final 10 years of simulations. The relatively small magnitude of global mean ΔT_a_ results from the contrasting warming and cooling effects in different grid cells (Fig. 1c–f). In fact, the spatial variations (standard deviation, SD) of ΔT_a_ range from 0.38 to 0.42 °C across different scenarios, much higher than the global mean ΔT_a_. Strong interannual variability (IAV) was also found in the last 20 years of the global mean ΔT_a_ (IAV = 0.16–0.19 °C, see details in Supplementary Methods 4.3, Supplementary Figs. 19 and 20).

Spatial patterns of ΔT_a_ differ among the four idealized bioenergy crop scenarios using the composite cultivation map (Fig. 1c–f). T_a_ changes more in cold regions than in warm regions (Fig. 1b and Supplementary Fig. 21). In boreal regions, all bioenergy crops exert a cooling effect on average (from 50°N to 80°N in Fig. 1b), mainly due to strong cooling signals in northern Europe and Siberia (Fig. 1c–f). In the warm northern temperate regions, the impacts of bioenergy crop plantation scenarios on T_a_ are very different (Fig. 1b). We find a cooling effect in the band from 30°N to 50°N for S_p&w_, with cooling epicenters in China, central Asia and the eastern US. In contrast, when switchgrass or eucalypt plantations are assumed (i.e., S_swi_ and S_euc_), we obtained warming effects in these regions (Fig. 1c, f). Across temperate Asia, the warming impact in S_euc_ (Fig. 1c) is stronger than in scenarios with herbaceous bioenergy crops (i.e., S_mis_ and S_swi_, Fig. 1e, f). In tropical regions, the magnitude of ΔT_a_ is relatively small for the scenarios based on the composite cultivation map (Fig. 1b). Compared to the herbaceous bioenergy crop scenarios (i.e., S_mis_ and S_swi_), the woody bioenergy crop scenarios (i.e., S_euc_ and S_p&w_) significantly cool the climate in regions of widespread cultivation, e.g., in southeast Brazil (Fig. 1c, d). Similarly, in sub-Saharan Africa, woody bioenergy crop scenarios have significant cooling signals, while temperature change from herbaceous bioenergy crops is weaker (Fig. 1c–f). Bioenergy crops have little impact on T_a_ in southeast Asia. A cooling effect is found in the north of Australia for all bioenergy scenarios using the composite cultivation map, while central and west Australia and New Zealand generally show warming effects, except in S_mis_ (Fig. 1c, d, f). By comparison, S_mis_ induces more cooling effects than warming effects in Oceania (Fig. 1e).

### Contribution of energy fluxes to ΔT_a_

We decomposed the ΔT_a_ induced by bioenergy crops into components related to the local surface energy balance and to the large-scale circulation^23,24^, based on:

1 $${\Delta {{{{{\rm{T}}}}}}}_{{{{{{\rm{a}}}}}}}=\Delta {{{{{{\rm{T}}}}}}}_{{{{{{\rm{a}}}}}}}^{{{{{{\rm{local}}}}}}}+\Delta {{{{{{\rm{T}}}}}}}_{{{{{{\rm{a}}}}}}}^{{{{{{\rm{cir}}}}}}}$$

where $\Delta {{{{{{\rm{T}}}}}}}_{{{{{{\rm{a}}}}}}}^{{{{{{\rm{local}}}}}}}$ is the temperature change induced by changes in the local surface energy balance (see details in Eqs. (13) and (14) of “Methods” and Supplementary Figs. 14–18). $\Delta {{{{{{\rm{T}}}}}}}_{{{{{{\rm{a}}}}}}}^{{{{{{\rm{cir}}}}}}}$ represents the impact of atmospheric circulation changes induced by changing the coverage of bioenergy crops (see details in “Methods” and Supplementary Methods 2.4). $\Delta {{{{{{\rm{T}}}}}}}_{{{{{{\rm{a}}}}}}}^{{{{{{\rm{local}}}}}}}$ can be determined from refs. ^23,24^:

2 $$\Delta {{{{{{\rm{T}}}}}}}_{{{{{{\rm{a}}}}}}}^{{{{{{\rm{local}}}}}}}=\frac{1}{{{{{{{\rm{f}}}}}}}_{{{{{{\rm{a}}}}}}}}\left[-{{{{{{\rm{SW}}}}}}}_{{{{{{\rm{d}}}}}}{{{{{\rm{n}}}}}}}\Delta {{\upalpha }}+\left(1-{{\upalpha }}\right)\Delta {{{{{{\rm{SW}}}}}}}_{{{{{{\rm{dn}}}}}}}-\Delta {{{{{\rm{LE}}}}}}-\Delta {{{{{\rm{G}}}}}} +\frac{{{\rho }}\cdot {{{{{{\rm{C}}}}}}}_{{{{{{\rm{p}}}}}}}}{{{{{{{\rm{r}}}}}}}_{{{{{{\rm{a}}}}}}}^{2}}\left({{{{{{\rm{T}}}}}}}_{{{{{{\rm{s}}}}}}}-{{{{{{\rm{T}}}}}}}_{{{{{{\rm{a}}}}}}}\right)\Delta {{{{{{\rm{r}}}}}}}_{{{{{{\rm{a}}}}}}}+{{{\varepsilon }}}_{{{{{{\rm{s}}}}}}}{{\upsigma }}{{{{{{\rm{T}}}}}}}_{{{{{{\rm{a}}}}}}}^{4}\Delta {{{\upvarepsilon }}}_{{{{{{\rm{a}}}}}}}\right]$$

where f_a_ is an energy redistribution factor (see “Methods”), and ∆SW_dn_, Δα, ∆LE, ∆G, ∆r_a_, and ∆ε_a_ are changes in surface downward shortwave radiation, surface albedo, latent heat flux, ground heat flux, aerodynamic resistance, and longwave radiative emissivity of air, respectively. C_p_, ρ, ε_s_, and σ are specific heat capacity of air, air density, surface longwave radiative emissivity (prescribed as 1.0 in the coupled model), and Stephan–Boltzmann constant (5.67 × 10^−8^ W·m^−2^·K^−4^), respectively.

Globally, for woody bioenergy crop scenarios based on the composite cultivation map (i.e., S_euc_ and S_p&w_), $\Delta {{{{{{\rm{T}}}}}}}_{{{{{{\rm{a}}}}}}}^{{{{{{\rm{local}}}}}}}$ is a net cooling effect with dominant contributions from decreased aerodynamic resistance (S_euc_) or increased albedo (S_p&w_). On the other hand, the sign of $\Delta {{{{{{\rm{T}}}}}}}_{{{{{{\rm{a}}}}}}}^{{{{{{\rm{cir}}}}}}}$ differs between S_euc_ (warming) and S_p&w_ (cooling) (Fig. 2a). Compared to S_p&w_, the cooling effect of $\Delta {{{{{{\rm{T}}}}}}}_{{{{{{\rm{a}}}}}}}^{{{{{{\rm{local}}}}}}}$ in S_euc_ is largely counteracted by the warming effect from $\Delta {{{{{{\rm{T}}}}}}}_{{{{{{\rm{a}}}}}}}^{{{{{{\rm{cir}}}}}}}$, leading to a smaller net ΔT_a_. In contrast, for herbaceous bioenergy crop scenarios, $\Delta {{{{{{\rm{T}}}}}}}_{{{{{{\rm{a}}}}}}}^{{{{{{\rm{local}}}}}}}$ is a net warming effect, while $\Delta {{{{{{\rm{T}}}}}}}_{{{{{{\rm{a}}}}}}}^{{{{{{\rm{cir}}}}}}}$ is a cooling effect (Fig. 2a). For both S_mis_ and S_swi_, the cooling effect of $\Delta {{{{{{\rm{T}}}}}}}_{{{{{{\rm{a}}}}}}}^{{{{{{\rm{cir}}}}}}}$ outweighs the warming effect of $\Delta {{{{{{\rm{T}}}}}}}_{{{{{{\rm{a}}}}}}}^{{{{{{\rm{local}}}}}}}$ causing a global net cooling effect (global mean ΔT_a_ < 0). The warming effect of $\Delta {{{{{{\rm{T}}}}}}}_{{{{{{\rm{a}}}}}}}^{{{{{{\rm{local}}}}}}}$ for the herbaceous bioenergy crop scenarios is mainly contributed by increased aerodynamic resistance, decreased latent heat and increased downward shortwave radiation. The global average cooling effect of albedo and ground heat flux and the warming effect of downward shortwave radiation are consistent among the four bioenergy crop scenarios based on the composite cultivation map (Fig. 2a).

Over the cultivated regions in the composite cultivation map, bioenergy crop cultivations show a cooling effect except for switchgrass (Fig. 2b). S_euc_, S_p&w_ and S_mis_ enhance local evapotranspiration and thus decrease ${{{{{{\rm{T}}}}}}}_{{{{{{\rm{a}}}}}}}^{{{{{{\rm{local}}}}}}}$ (Fig. 2b and Supplementary Figs. 14–17), whereas S_swi_ decreases evapotranspiration which offsets the cooling effects from other terms in Eq. (2) (e.g., a higher albedo), resulting in an increased $\Delta {{{{{{\rm{T}}}}}}}_{{{{{{\rm{a}}}}}}}^{{{{{{\rm{local}}}}}}}$ (Fig. 2b and Supplementary Fig. 15). All the idealized bioenergy crop scenarios using the composite cultivation map reduce the aerodynamic resistance which induces a cooling effect where they are cultivated. In addition, the magnitudes of the different gross components in Eq. (2) are much larger for woody bioenergy crops than for herbaceous crops over the cultivated regions (Fig. 2b), due to the differences in vegetation properties (e.g., LAI and roughness) and changes in the related variables (e.g., wind speed, humidity, and cloud) between woody and herbaceous crops (Supplementary Figs. 22 and 23, see details in Supplementary Discussion 1).

Outside the regions of bioenergy crop cultivation, $\Delta {{{{{{\rm{T}}}}}}}_{{{{{{\rm{a}}}}}}}^{{{{{{\rm{local}}}}}}}$ shows warming effects for all the idealized bioenergy crop scenarios based on the composite cultivation map (Fig. 2c), while $\Delta {{{{{{\rm{T}}}}}}}_{{{{{{\rm{a}}}}}}}^{{{{{{\rm{cir}}}}}}}$ has cooling effects. Atmospheric circulation redistributes surface energy changes^23,28,29^, and wind speed and surface roughness height are tightly associated with atmospheric circulation (Supplementary Fig. 24). Therefore, outside the cultivated regions in the composite cultivation map, where there is no land cover change in the bioenergy crop scenarios (same as S_ref_), atmospheric circulation and teleconnections alter local radiative forcing indirectly through exchanges of heat between different regions (Supplementary Figs. 14–17). Changes in local radiative forcing further influence vegetation gas exchange and structure and thus the surface energy balance, e.g., changes in downward shortwave radiation, air emissivity and aerodynamic resistance (Fig. 2c and Supplementary Figs. 12–15). This is supported by the fact that LAI and radiative forcing are slightly changed outside the regions of bioenergy crop cultivation (Supplementary Fig. 24 and Fig. 2c).

### T_a_ changes at continental scale

**Fig. 3:**
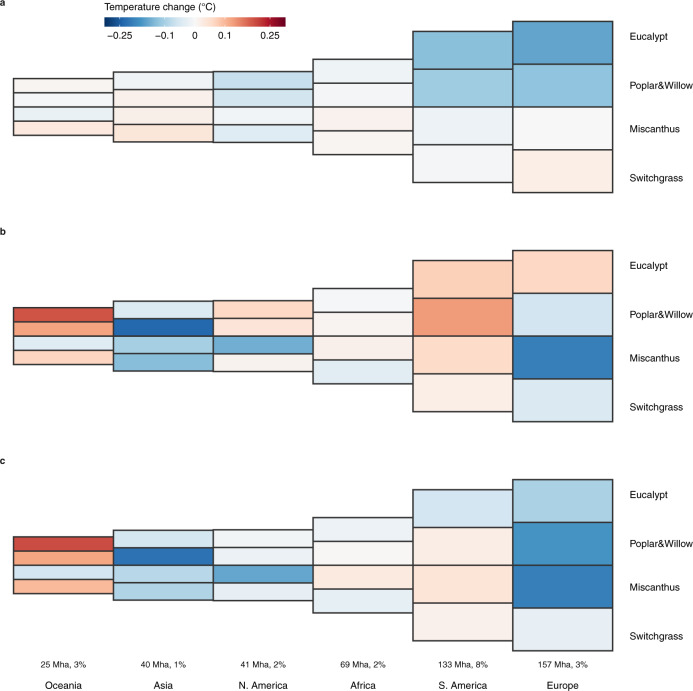
Bioenergy crop cultivation area and mean temperature change in each continent. Air temperature changes induced by altered local surface energy balance (**a**) and by atmospheric circulation (**b**) and the net air temperature change (**c**) are the average values over the whole continent. Sizes of rectangles represent relative bioenergy crop cultivation areas in different continents in the composite cultivation map, and color gradients indicate temperature change. Numbers show the area of bioenergy crop cultivation in each continent (left, M ha) in the composite cultivation map and its proportion of the total continental area (right, %).

Based on the composite bioenergy crop cultivation map, the effects of local surface energy balance changes in air temperature ($\Delta {{{{{{\rm{T}}}}}}}_{{{{{{\rm{a}}}}}}}^{{{{{{\rm{local}}}}}}}$) range from warming in Oceania, which has the smallest bioenergy crop cultivation area, to cooling in Europe, which has the largest area of bioenergy crop cultivation (Fig. 3a). In fact, $\Delta {{{{{{\rm{T}}}}}}}_{{{{{{\rm{a}}}}}}}^{{{{{{\rm{local}}}}}}}$ is significantly correlated with the area of bioenergy crop cultivation in the composite cultivation map across different continents (*p* < 0.05) for woody bioenergy crop scenarios (Supplementary Fig. 25). However, changes in atmospheric circulation ($\Delta {{{{{{\rm{T}}}}}}}_{{{{{{\rm{a}}}}}}}^{{{{{{\rm{cir}}}}}}}$, Fig. 3b) that redistribute energy spatially show a more subtle pattern (Fig. 3b), either enhancing or weakening the $\Delta {{{{{{\rm{T}}}}}}}_{{{{{{\rm{a}}}}}}}^{{{{{{\rm{local}}}}}}}$ signal. As a consequence, mean ΔT_a_ at the continental scale depends on both $\Delta {{{{{{\rm{T}}}}}}}_{{{{{{\rm{a}}}}}}}^{{{{{{\rm{local}}}}}}}$ and $\Delta {{{{{{\rm{T}}}}}}}_{{{{{{\rm{a}}}}}}}^{{{{{{\rm{cir}}}}}}}$ as well as on the bioenergy crop type considered. Therefore, continents with a larger area of bioenergy crop cultivation do not always show the stronger cooling effect (Fig. 3c and Supplementary Fig. 25).

Woody bioenergy crop cultivation largely decreases ${{{{{{\rm{T}}}}}}}_{{{{{{\rm{a}}}}}}}^{{{{{{\rm{local}}}}}}}$ in Europe ($\Delta {{{{{{\rm{T}}}}}}}_{{{{{{\rm{a}}}}}}}^{{{{{{\rm{local}}}}}}}$ = −0.16 and −0.12 °C for S_euc_ and S_p&w_, Fig. 3a and Supplementary Fig. 26) due to the effects of decreased aerodynamic resistance, increased evapotranspiration, and decreased downward shortwave radiation (Supplementary Fig. 26). The continental mean ΔT_a_ is also contributed by $\Delta {{{{{{\rm{T}}}}}}}_{{{{{{\rm{a}}}}}}}^{{{{{{\rm{cir}}}}}}}$ (0.06 and −0.06 °C for S_euc_ and S_p&w_, Fig. 3b and Supplementary Fig. 26). In South America, which has substantial areas of bioenergy crop cultivation in our scenarios (133 M ha), the cooling effect of $\Delta {{{{{{\rm{T}}}}}}}_{{{{{{\rm{a}}}}}}}^{{{{{{\rm{local}}}}}}}$ is counteracted by a warming effect of $\Delta {{{{{{\rm{T}}}}}}}_{{{{{{\rm{a}}}}}}}^{{{{{{\rm{cir}}}}}}}$, leading to a net increase of T_a_ in all scenarios based on the composite cultivation map except S_euc_ (Fig. 3 and Supplementary Fig. 26). Bioenergy crop scenarios generally result in weak effects on the mean T_a_ of Africa, with all components contributing little to ΔT_a_ (Supplementary Fig. 26). Asia and North America have similar bioenergy crop cultivation areas (40 and 41 M ha, respectively) and both show net cooling effects for all bioenergy crop scenarios, but the signs of $\Delta {{{{{{\rm{T}}}}}}}_{{{{{{\rm{a}}}}}}}^{{{{{{\rm{local}}}}}}}$ and $\Delta {{{{{{\rm{T}}}}}}}_{{{{{{\rm{a}}}}}}}^{{{{{{\rm{cir}}}}}}}$ are generally opposite in these two continents (Fig. 3 and Supplementary Fig. 26). In contrast, in Oceania, which has the smallest bioenergy crop cultivation areas, all bioenergy crop scenarios using the composite cultivation map, with the exception of S_mis_, show warming effects (ΔT_a_ > 0) contributed by positive $\Delta {{{{{{\rm{T}}}}}}}_{{{{{{\rm{a}}}}}}}^{{{{{{\rm{cir}}}}}}}$ (Supplementary Fig. 26).

In addition, although mean T_a_ over the regions of bioenergy crop cultivation based on the composite cultivation map within each continent generally decreases due to reduced aerodynamic resistance and enhanced latent heat, especially for woody bioenergy crops (Supplementary Fig. 27), the continental mean ΔT_a_ is mainly contributed by ΔT_a_ outside the cultivated regions, implying the importance of the teleconnection of temperature changes (Supplementary Fig. 28). For example, poplar & willow cultivation decreases the mean T_a_ over their cultivated regions in all continents (Supplementary Fig. 27), but this signal is offset by teleconnection warming outside the cultivated regions in Oceania, Africa, and South America (Supplementary Fig. 28), resulting in a positive mean ΔT_a_ for the whole continent (Fig. 3c and Supplementary Fig. 26). Despite contrasting changes in T_a_ within and outside the bioenergy crop cultivation regions in the composite cultivation map, the mean ΔT_a_ and the contributions of various energy fluxes for the whole continent (Supplementary Fig. 26) generally agree with the changes in the grid cells with significant ΔT_a_ (grid cells with black shading in Fig. 1c–f), but the former magnitudes are smaller (Supplementary Figs. 26 and 29).

### Sensitivity tests of additional scenarios

**Fig. 4:**
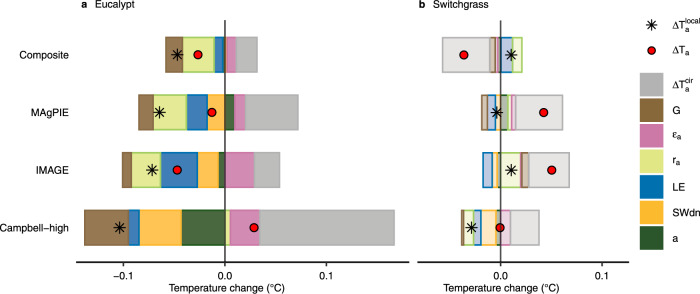
Contributions of different components to air temperature changes at the global scale using different cultivation maps. Two panels represent eucalypt cultivations (**a**) and switchgrass cultivation (**b**). Four rows of bars represent four cultivation maps (i.e., the composite, MAgPIE, IMAGE, and Campbell-high map). Bars for$\Delta {{{{{{\rm{T}}}}}}}_{{{{{{\rm{a}}}}}}}^{{{{{{\rm{cir}}}}}}}$, G, ε_a_, r_a_, LE, SW_dn_, and α represent different components that contribute to the air temperature change, i.e., the altered atmospheric circulation, ground heat flux, longwave radiative emissivity of air, aerodynamic resistance, latent heat flux, surface downward shortwave radiation, and surface albedo, respectively. The net air temperature change is shown by red dots, which is the sum of all bars. Black asterisks represent the contribution of altered local surface energy balance, which is the sum of the colored bars excluding the gray bar.

To quantify the sensitivity of biophysical climate effects to the choice of a bioenergy crop cultivation map, we performed six additional experimental simulations (Supplementary Methods 4.2), comprising three individual cultivation maps (two IAM maps^17^ from IMAGE and MAgPIE, and one marginal land map from Campbell et al.^14^, namely Campbell-high, Supplementary Fig. 10a, c, d) and two representative bioenergy crop types (eucalypt and switchgrass). In the additional simulations based on the three individual cultivation maps and two bioenergy crop types, the global net ΔT_a_ (averaged over the last 10 years) ranges from −0.05 to 0.05 °C across various simulations with significant ΔT_a_ appearing in 9–25% of the global lands (Fig. 4 and Supplementary Fig. 30). Similar to the results from the composite map (Fig. 1), ΔT_a_ also has strong spatial heterogeneity (spatial SD ranges from 0.25 to 0.37, Supplementary Fig. 30) and high interannual variability (20-year IAV ranges from 0.15 to 0.2 °C across different scenarios).

At the global scale, the signs of $\Delta {{{{{{\rm{T}}}}}}}_{{{{{{\rm{a}}}}}}}^{{{{{{\rm{local}}}}}}}$ and most energy components for eucalypt are robust among all cultivation maps, but the magnitudes vary, especially for $\Delta {{{{{{\rm{T}}}}}}}_{{{{{{\rm{a}}}}}}}^{{{{{{\rm{cir}}}}}}}$ (Fig. 4). Different from the net cooling effect of ΔT_a_ using composite, IMAGE, or MAgPIE map, the positive ΔT_a_ in ${{{{{{\rm{S}}}}}}}_{{{{{{\rm{euc}}}}}}}^{{{{{{\rm{Cam}}}}}}}$ (i.e., the simulation using the Campbell-high map for eucalypt) is caused by the large warming effect of $\Delta {{{{{{\rm{T}}}}}}}_{{{{{{\rm{a}}}}}}}^{{{{{{\rm{cir}}}}}}}$, which offsets the cooling effect of $\Delta {{{{{{\rm{T}}}}}}}_{{{{{{\rm{a}}}}}}}^{{{{{{\rm{local}}}}}}}$. By contrast, the changes of biophysical effects of switchgrass are different among simulations with different cultivation maps. ΔT_a_ shows a cooling effect in S_swi_, but a warming effect in ${{{{{{\rm{S}}}}}}}_{{{{{{\rm{swi}}}}}}}^{{{{{{\rm{IMA}}}}}}}$ and ${{{{{{\rm{S}}}}}}}_{{{{{{\rm{swi}}}}}}}^{{{{{{\rm{MAg}}}}}}}$, and a nearly neutral effect in ${{{{{{\rm{S}}}}}}}_{{{{{{\rm{swi}}}}}}}^{{{{{{\rm{Cam}}}}}}}$. Compared to eucalypt, the responses of each component to switchgrass cultivations are relatively small using all cultivation maps, resulting in higher sensitivity of ΔT_a_ to cultivation maps. The signs of ΔT_a_ for switchgrass are largely determined by $\Delta {{{{{{\rm{T}}}}}}}_{{{{{{\rm{a}}}}}}}^{{{{{{\rm{cir}}}}}}}$.

Similarly, at the continental scale, the signal of $\Delta {{{{{{\rm{T}}}}}}}_{{{{{{\rm{a}}}}}}}^{{{{{{\rm{local}}}}}}}$ for eucalypt is generally robust with respect to the choice of a cultivation map, showing consistent patterns between the idealized composite maps and the three individual maps in most continents, but the signs of ΔT_a_ also depend on the $\Delta {{{{{{\rm{T}}}}}}}_{{{{{{\rm{a}}}}}}}^{{{{{{\rm{cir}}}}}}}$ (Supplementary Fig. 31). In agreement with the differences between eucalypt and switchgrass at the global scale (Fig. 4), the magnitudes of biophysical effect change for switchgrass are also lower than eucalypt at the continental scale, and the patterns are more diverse across various maps (Supplementary Fig. 31 and 32). Only $\Delta {{{{{{\rm{T}}}}}}}_{{{{{{\rm{a}}}}}}}^{{{{{{\rm{local}}}}}}}$ in Europe and $\Delta {{{{{{\rm{T}}}}}}}_{{{{{{\rm{a}}}}}}}^{{{{{{\rm{cir}}}}}}}$ in Oceania and Africa are consistent for switchgrass cultivation across the four maps (one composite and three individual maps). Combining $\Delta {{{{{{\rm{T}}}}}}}_{{{{{{\rm{a}}}}}}}^{{{{{{\rm{local}}}}}}}$ and $\Delta {{{{{{\rm{T}}}}}}}_{{{{{{\rm{a}}}}}}}^{{{{{{\rm{cir}}}}}}}$, however, no consistent ΔT_a_ for switchgrass were found in any continent (Supplementary Fig. 32).

The original land use types upon which bioenergy crop cultivation is expanded (Supplementary Fig. 10) also determine the changes in the surface energy budget. Cultivation regions from the MAgPIE and Campbell-high maps were generally converted from short vegetation like cropland and grassland, while in the IMAGE map, 78% of the bioenergy cultivation lands were converted from forest. Even so, both $\Delta {{{{{{\rm{T}}}}}}}_{{{{{{\rm{a}}}}}}}^{{{{{{\rm{local}}}}}}}$ and ΔT_a_ in ${{{{{{\rm{S}}}}}}}_{{{{{{\rm{euc}}}}}}}^{{{{{{\rm{IMA}}}}}}}$ are negative (Fig. 4), indicating the stronger cooling effects of eucalypt plantations than previous forest types. Compared to S_euc_ and ${{{{{{\rm{S}}}}}}}_{{{{{{\rm{euc}}}}}}}^{{{{{{\rm{MAg}}}}}}}$, where eucalypt was cultivated on short-vegetation lands, the cooling effects in ${{{{{{\rm{S}}}}}}}_{{{{{{\rm{euc}}}}}}}^{{{{{{\rm{IMA}}}}}}}$ is even stronger (Fig. 4), probably due to the larger cultivation area in the IMAGE map (523 M ha) than the composite and MAgPIE maps (466 and 408 M ha, Supplementary Fig. 10). By contrast, replacing forests with switchgrass in ${{{{{{\rm{S}}}}}}}_{{{{{{\rm{swi}}}}}}}^{{{{{{\rm{IMA}}}}}}}$, both $\Delta {{{{{{\rm{T}}}}}}}_{{{{{{\rm{a}}}}}}}^{{{{{{\rm{local}}}}}}}$ and ΔT_a_ show warming effects ($\Delta {{{{{{\rm{T}}}}}}}_{{{{{{\rm{a}}}}}}}^{{{{{{\rm{local}}}}}}}$ = 0.01 °C, ΔT_a_ = 0.05 °C). The Campbell-high map represents a widely spreading BECCS cultivation scenario, and its biophysical effects are rather different from those with cultivation areas more concentrated in a few regions (e.g., the larger $\Delta {{{{{{\rm{T}}}}}}}_{{{{{{\rm{a}}}}}}}^{{{{{{\rm{cir}}}}}}}$ in the eucalypt cultivation scenario based on the Campbell-high map, see Fig. 4a and Supplementary Discussion 2). This illustrates the importance of the spatial cultivation patterns on the biophysical effects. Note the differences in biophysical effects between the composite map and the three individual maps also include the differences between the two reference simulations (see “Methods” and Supplementary Discussion 3). For example, the large difference of ΔT_a_ between the three individual maps and the idealized composite map in the boreal regions (Supplementary Fig. 30) is mainly caused by the difference between the two reference simulations (Supplementary Fig. 33).

## Discussion

We found that, even though the cultivation area occupies 3.8% ± 0.5% of the global total land area, bioenergy crops exert strong regional biophysical effects, leading to a global net change in air temperature of −0.08 ~ +0.05 °C. The CDR target of the BECCS scenarios used in IAMs is 128 PgC till 2100 for RCP 2.6^6^, approximately corresponding to a cooling effect of 0.09 ~ 0.32 °C (Supplementary Table 5). Thus, the biophysical cooling or warming effects of bioenergy crop cultivation can significantly strengthen or weaken the effectiveness of BECCS in limiting the temperature increments depending on the cultivation map and the bioenergy crop type. However, the temperature changes in the bioenergy crop scenarios do have very large spatial variations and important climate teleconnections to other areas of the globe (Fig. 1c–f). For example, from the four idealized bioenergy crop scenarios based on the composited cultivation map, warming effects in Alaska and northwestern Canada may cause greenhouse gas release from thawing permafrost^30,31^, while strong cooling effects in Eurasia, between 60°N and 80°N, may protect permafrost from thawing or reduce methane emissions from wetlands. Therefore, the biophysical effects of bioenergy crop cultivation do not only alter global temperature directly, but also induce secondary effects on natural greenhouse gas fluxes, which should also be taken into account when considering large-scale BECCS deployment. Meanwhile, altered atmospheric circulation is a major contributor to the net change in global air temperature, but its spatial variations may rely on the choice of the atmosphere model, and the relevant uncertainties need further investigation.

In addition, the six additional scenarios using three plausible cultivation maps and two representative bioenergy crops demonstrated the importance of the crop type choice, the original land use type upon which bioenergy crop are expanded, the total cultivation area and its spatial distribution patterns. Cultivating eucalypt shows generally cooling effects from ΔT_a_ and $\Delta {{{{{{\rm{T}}}}}}}_{{{{{{\rm{a}}}}}}}^{{{{{{\rm{local}}}}}}}$ (Fig. 4), which are more robust than if switchgrass is used as the main bioenergy crop across various cultivation maps, implying that eucalypt is superior to switchgrass in cooling the lands biophysically. Replacing forests with switchgrass in ${{{{{{\rm{S}}}}}}}_{{{{{{\rm{swi}}}}}}}^{{{{{{\rm{IMA}}}}}}}$ not only results in biophysical warming effects (Fig. 4) but could also release more carbon through deforestation than converting other short vegetation to bioenergy crops. Thus, deforestation should be avoided. The magnitude of changes in the biophysical effects also depends on the total cultivation area. With the largest cultivation areas in the IMAGE map, ΔT_a_ shows greatest cooling effects for eucalypt and greatest warming effects for switchgrass among the four maps (Fig. 4). Last, cultivation concentrated in a few regions rather than spreading fragmented cultivation (like the spatial cultivation patterns in the Campbell-high map) is recommended, which is also conducive to land management.

Air temperature changes in different continents are disproportionate to their areas of bioenergy crop cultivation. In the composite cultivation map, the bioenergy crop cultivation area in Europe (157 M ha) is about four times larger than that in Asia (40 M ha), but both continents have similar cooling magnitudes for all four idealized bioenergy crop scenarios. In other continents, air temperature changes depend on bioenergy crop types. Therefore, there is a requirement for new trans-regional metrics to appreciate the climate impacts of BECCS and for international collaboration to share the responsibility of bioenergy crop cultivation and to address the challenges inherent in slowing down global warming by considering both the biogeochemical and biophysical effects of climate mitigation options. In addition to the biophysical effects, the feasibility of BECCS also relies on other dimensions like the related land use change carbon emissions^6^, the water demand^9,10^ and the nutrient cycle^11–13^. A full assessment of the nexus of these aspects is thus needed.

## Methods

### Model and simulations

To simulate the biophysical feedbacks of large-scale bioenergy crop cultivation, ORCHIDEE-MICT-BIOENERGY was coupled with the atmospheric general circulation model (LMD_Z_)^32,33^ of the Institute Pierre Simon Laplace climate model (IPSL-CM^25^, see Supplementary Methods 1 and 2 for details). The ocean model was excluded from the coupling, and sea surface temperature (SST) was prescribed using climatology data with seasonal variations (from the Atmospheric Model Intercomparison Project (AMIP), http://www-pcmdi.llnl.gov/projects/amip). The spatial resolution of the coupled model was 1.26° latitude × 2.5° longitude.

ORCHIDEE-MICT-BIOENERGY was developed specifically to represent bioenergy crop modeling^26^ based on ORCHIDEE-MICT^34^. Two woody and two herbaceous bioenergy crop types were implemented: (1) eucalypts, (2) poplar & willow (treated as the same PFT), (3) miscanthus, and (4) switchgrass. ORCHIDEE-MICT-BIOENERGY includes specific biomass harvest modules for bioenergy crops: annual harvest of herbaceous crops and the periodic harvest of woody crops after each 5-year rotation. Parameters related to critical processes of bioenergy crops, i.e., photosynthesis, carbon allocation, phenology, and biomass harvest were systematically calibrated based on field data^26,27^. The model is capable of capturing the growth dynamics of bioenergy crops and reproducing the biomass yields in a large global yield observation dataset^26,27^. The LMDz used here is version 6 of the Laboratoire de Météorologie Dynamique atmospheric general circulation model with zooming capability^32,33^. It simulates the fundamental dynamical and physical processes of the atmosphere (e.g., radiation, moist convection, cloud macro-, and micro-physical properties, etc.) using 79 layers in the vertical.

Based on the composited cultivation map, we performed five 50-year coupled simulations starting from 2015: a reference scenario (S_ref_) and four idealized bioenergy crop scenarios with cultivation of different bioenergy crops (S_euc_: eucalypt, S_p&w_: poplar & willow, S_mis_: miscanthus, S_swi_: switchgrass, see Supplementary Methods 4.1). In S_ref_, the BECCS regions (Fig. 1a, Supplementary Methods 3) were covered by generic grain crops because the BECCS regions in the composite cultivation map were mainly abandoned cropland. In each of the four bioenergy crop scenarios, the BECCS regions in the composite cultivation map were covered only with the corresponding bioenergy crop type. At the end of the 50-year simulations, the key vegetation features (e.g., photosynthesis and leaf area index) that are closely related to the energy budget (e.g., evapotranspiration and albedo) reach a steady state, implying that the simulation length is sufficient to derive a robust signal of biophysical effects from bioenergy crop (see details in Supplementary Methods 4.3 and Supplementary Figs. 12 and 13).

We also performed seven additional simulations to analyze the sensitivity of biophysical effects to different cultivation maps. Seven additional simulations include one reference scenario (${{{{{{\rm{S}}}}}}}_{{{{{{\rm{ref}}}}}}}^{{{{{{\rm{pre}}}}}}}$) and six more realistic bioenergy crop scenarios (i.e., 3 individual cultivation maps × 2 representative bioenergy crop types, Supplementary Methods 4.2 and Supplementary Table 4). In these three individual cultivation maps, the bioenergy crop coverage is fractional in each grid cell (Supplementary Fig. 10a, c, d), which is considered more realistic than in the composite map where entire grid cells in the BECCS regions were fully covered by bioenergy crops (Supplementary Fig. 10f). A corresponding reference simulation (${{{{{{\rm{S}}}}}}}_{{{{{{\rm{ref}}}}}}}^{{{{{{\rm{pre}}}}}}}$) was also run to match the six additional simulations. Different from S_ref_ with food crop in the bioenergy crop cultivation grid cells, ${{{{{{\rm{S}}}}}}}_{{{{{{\rm{ref}}}}}}}^{{{{{{\rm{pre}}}}}}}$ used the present-day observed fractional land covers in each grid cell (Supplementary Methods 4.2).

For simulations using either the composite map or the individual cultivation maps, the differences in the outputs between the reference and bioenergy crop scenarios were analyzed to determine the influence of each bioenergy crop cultivation. Here, we mainly focus on the spatial patterns of bioenergy cultivation impacts, and thus we used the outputs averaged over the last 10 years of each scenario, covering two 5-year rotation cycles set in the model for woody bioenergy crop harvest.

### Decomposing ΔT_a_ into different factors

Changes of air temperature at 2-m height (ΔT_a_) in response to bioenergy crop cultivation were decomposed into components that represent key processes and mechanisms of air temperature change following the methods of Li et al.^24^ and Zeng et al.^23^. The decomposition is fundamentally based on the land-surface energy balance:

3 $${{{{{{\rm{SW}}}}}}}_{{{{{{\rm{dn}}}}}}}-{{{{{\rm{S}}}}}}{{{{{{\rm{W}}}}}}}_{{{{{{{\rm{u}}}}}}}_{{{{{{\rm{P}}}}}}}}+{{{{{\rm{L}}}}}}{{{{{{\rm{W}}}}}}}_{{{{{{\rm{d}}}}}}{{{{{\rm{n}}}}}}}-{{{{{\rm{L}}}}}}{{{{{{\rm{W}}}}}}}_{{{{{{\rm{up}}}}}}}={{{{{\rm{LE}}}}}}+{{{{{\rm{H}}}}}}+{{{{{\rm{G}}}}}}$$

where SW_dn_, SW_up_, LW_dn_, and LW_up_, denote downward shortwave radiation, upward shortwave radiation, downward longwave radiation, and upward longwave radiation, respectively. LE, H, and G represent the latent, sensible, and ground heat fluxes, respectively.

Net shortwave radiation at the surface (SW_dn_−SW_up_) can be expressed as a combination of surface albedo (α) and downward shortwave radiation (SW_dn_):

4 $${{{{{{\rm{SW}}}}}}}_{{{{{{\rm{dn}}}}}}}-{{{{{\rm{S}}}}}}{{{{{{\rm{W}}}}}}}_{{{{{{{\rm{u}}}}}}}_{{{{{{\rm{P}}}}}}}}={{{{{{\rm{SW}}}}}}}_{{{{{{\rm{dn}}}}}}}\,\left(1-{{\alpha }}\right)$$

Net longwave radiation at the surface (LW_dn_−LW_up_) can be expressed as a function of air temperature (T_a_), surface temperature (T_s_), and the longwave radiative emissivity of air (ε_a_) according to the Stephan–Boltzmann law:

5 $${{{{{\rm{L}}}}}}{{{{{{\rm{W}}}}}}}_{{{{{{\rm{d}}}}}}{{{{{\rm{n}}}}}}}-{{{{{\rm{L}}}}}}{{{{{{\rm{W}}}}}}}_{{{{{{\rm{up}}}}}}}={{{\varepsilon }}}_{{{{{{\rm{s}}}}}}}{{\sigma }}\left({{{\varepsilon }}}_{{{{{{\rm{a}}}}}}}{{{{{{\rm{T}}}}}}}_{{{{{{\rm{a}}}}}}}^{4}-{{{{{{\rm{T}}}}}}}_{{{{{{\rm{s}}}}}}}^{4}\right)$$

where ε_s_ is surface longwave radiative emissivity (prescribed as 1.0 in the coupled model) and σ is the Stephan–Boltzmann constant (5.67 × 10^−8^ W·m^−2^·K^−4^).

Sensible heat flux (H) can be given by:

6 $${{{{{\rm{H}}}}}}={{\rho }}\cdot {{{{{{\rm{C}}}}}}}_{{{{{{\rm{P}}}}}}}\frac{{{{{{{\rm{T}}}}}}}_{{{{{{\rm{s}}}}}}}-{{{{{{\rm{T}}}}}}}_{{{{{{\rm{a}}}}}}}}{{{{{{{\rm{r}}}}}}}_{{{{{{\rm{a}}}}}}}}$$

where r_a_, ρ, and C_P_ represent aerodynamic resistance, air density, and the specific heat capacity of air, respectively.

Based on Eqs. (4)–(6), Eq. (3) can be re-written as:

7 $${{{\varepsilon }}}_{{{{{{\rm{s}}}}}}}{{\sigma }}{{{{{{\rm{T}}}}}}}_{{{{{{\rm{s}}}}}}}^{4}+\frac{{{\rho }}{{{{{{\rm{C}}}}}}}_{{{{{{\rm{P}}}}}}}}{{{{{{{\rm{r}}}}}}}_{{{{{{\rm{a}}}}}}}}{{{{{{\rm{T}}}}}}}_{{{{{{\rm{s}}}}}}}={{{{{{\rm{SW}}}}}}}_{{{{{{\rm{dn}}}}}}}\,\left(1-{{\alpha }}\right)-{{{{{\rm{LE}}}}}}-{{{{{\rm{G}}}}}}+{{{\varepsilon }}}_{{{{{{\rm{s}}}}}}}{{\sigma }}{{{\varepsilon }}}_{{{{{{\rm{a}}}}}}}{{{{{{\rm{T}}}}}}}_{{{{{{\rm{a}}}}}}}^{4}+\frac{{{\rho }}{{{{{{\rm{C}}}}}}}_{{{{{{\rm{P}}}}}}}}{{{{{{{\rm{r}}}}}}}_{{{{{{\rm{a}}}}}}}}{{{{{{\rm{T}}}}}}}_{{{{{{\rm{a}}}}}}}$$

Differentiation with respect to T_s_ gives:

8 $${\triangle {{{{{\rm{T}}}}}}}_{{{{{{\rm{s}}}}}}}=\frac{1}{{{{{{{\rm{f}}}}}}}_{{{{{{\rm{s}}}}}}}}\left[-{{{{{{\rm{SW}}}}}}}_{{{{{{\rm{d}}}}}}{{{{{\rm{n}}}}}}}\Delta {{\alpha }}+\left(1-{{\alpha }}\right)\Delta {{{{{{\rm{SW}}}}}}}_{{{{{{\rm{dn}}}}}}}-\Delta {{{{{\rm{LE}}}}}}-\Delta {{{{{\rm{G}}}}}}+\frac{{{\rho }}\cdot {{{{{{\rm{C}}}}}}}_{{{{{{\rm{p}}}}}}}}{{{{{{{\rm{r}}}}}}}_{{{{{{\rm{a}}}}}}}^{2}}\left({{{{{\rm{T}}}}}}_{{{{{\rm{s}}}}}}-{{{{{{\rm{T}}}}}}}_{{{{{{\rm{a}}}}}}}\right)\Delta {{{{{{\rm{r}}}}}}}_{{{{{{\rm{a}}}}}}}+{{{\varepsilon }}}_{{{{{{\rm{s}}}}}}}{{\sigma }}{{{{{{\rm{T}}}}}}}_{{{{{{\rm{a}}}}}}}^{4}\Delta {{{\varepsilon }}}_{{{{{{\rm{a}}}}}}}\right]+\frac{{{{{{{\rm{f}}}}}}}_{{{{{{\rm{a}}}}}}}}{{{{{{{\rm{f}}}}}}}_{{{{{{\rm{s}}}}}}}}{\triangle {{{{{\rm{T}}}}}}}_{{{{{{\rm{a}}}}}}}$$

where f_s_ and f_a_ are the energy redistribution factors for surface temperature and air temperature:

9 $${{{{{{\rm{f}}}}}}}_{{{{{{\rm{s}}}}}}}=4{{{\varepsilon }}}_{{{{{{\rm{s}}}}}}}{{\sigma }}{{{{{{\rm{T}}}}}}}_{{{{{{\rm{s}}}}}}}^{3}+\frac{{{\rho }}{{{{{{\rm{C}}}}}}}_{{{{{{\rm{P}}}}}}}}{{{{{{{\rm{r}}}}}}}_{{{{{{\rm{a}}}}}}}}$$

10 $${{{{{{\rm{f}}}}}}}_{{{{{{\rm{a}}}}}}}=4{{{\varepsilon }}}_{{{{{{\rm{s}}}}}}}{{\sigma }}{{{\varepsilon }}}_{{{{{{\rm{a}}}}}}}{{{{{{\rm{T}}}}}}}_{{{{{{\rm{a}}}}}}}^{3}+\frac{{{\rho }}{{{{{{\rm{C}}}}}}}_{{{{{{\rm{P}}}}}}}}{{{{{{{\rm{r}}}}}}}_{{{{{{\rm{a}}}}}}}}$$

ΔT_a_ can be partitioned into changes in the local surface energy balance ($\Delta {{{{{{\rm{T}}}}}}}_{{{{{{\rm{a}}}}}}}^{{{{{{\rm{local}}}}}}}$) and changes in atmospheric circulation ($\Delta {{{{{{\rm{T}}}}}}}_{{{{{{\rm{a}}}}}}}^{{{{{{\rm{cir}}}}}}}$)^23,24^:

11 $$\Delta {{{{{{\rm{T}}}}}}}_{{{{{{\rm{a}}}}}}}=\Delta {{{{{{\rm{T}}}}}}}_{{{{{{\rm{a}}}}}}}^{{{{{{\rm{local}}}}}}}+\Delta {{{{{{\rm{T}}}}}}}_{{{{{{\rm{a}}}}}}}^{{{{{{\rm{cir}}}}}}}$$

Similarly, ${\triangle {{{{{\rm{T}}}}}}}_{{{{{{\rm{s}}}}}}}$ can be attributed to $\Delta {{{{{{\rm{T}}}}}}}_{{{{{{\rm{s}}}}}}}^{{{{{{\rm{local}}}}}}}$ and $\Delta {{{{{{\rm{T}}}}}}}_{{{{{{\rm{s}}}}}}}^{{{{{{\rm{cir}}}}}}}$:

12 $$\Delta {{{{{{\rm{T}}}}}}}_{{{{{{\rm{s}}}}}}}=\Delta {{{{{{\rm{T}}}}}}}_{{{{{{\rm{s}}}}}}}^{{{{{{\rm{local}}}}}}}+\Delta {{{{{{\rm{T}}}}}}}_{{{{{{\rm{s}}}}}}}^{{{{{{\rm{cir}}}}}}}$$

The first six terms on the right hand side of Eq. (8) constitute $\Delta {{{{{{\rm{T}}}}}}}_{{{{{{\rm{s}}}}}}}^{{{{{{\rm{local}}}}}}}$:

13 $$\Delta {{{{{{\rm{T}}}}}}}_{{{{{{\rm{s}}}}}}}^{{{{{{\rm{local}}}}}}}=\frac{1}{{{{{{{\rm{f}}}}}}}_{{{{{{\rm{s}}}}}}}}\left[-{{{{{{\rm{SW}}}}}}}_{{{{{{\rm{d}}}}}}{{{{{\rm{n}}}}}}}\Delta {{\alpha }}+\left(1-{{\alpha }}\right)\Delta {{{{{{\rm{SW}}}}}}}_{{{{{{\rm{dn}}}}}}}-\Delta {{{{{\rm{LE}}}}}}-\Delta {{{{{\rm{G}}}}}}+\frac{{{\rho }}\cdot {{{{{{\rm{C}}}}}}}_{{{{{{\rm{p}}}}}}}}{{{{{{{\rm{r}}}}}}}_{{{{{{\rm{a}}}}}}}^{2}}\left({{{{{{\rm{T}}}}}}}_{{{{{{\rm{s}}}}}}}-{{{{{{\rm{T}}}}}}}_{{{{{{\rm{a}}}}}}}\right)\Delta {{{{{{\rm{r}}}}}}}_{{{{{{\rm{a}}}}}}}+{{{\varepsilon }}}_{{{{{{\rm{s}}}}}}}{{\sigma }}{{{{{{\rm{T}}}}}}}_{{{{{{\rm{a}}}}}}}^{4}\Delta {{{\varepsilon }}}_{{{{{{\rm{a}}}}}}}\right].$$

$\Delta {{{{{{\rm{T}}}}}}}_{{{{{{\rm{s}}}}}}}^{{{{{{\rm{local}}}}}}}$ is further converted to $\Delta {{{{{{\rm{T}}}}}}}_{{{{{{\rm{a}}}}}}}^{{{{{{\rm{local}}}}}}}$ by Li et al.^24^ and Zeng et al.^23^:

14 $$\Delta {{{{{{\rm{T}}}}}}}_{{{{{{\rm{a}}}}}}}^{{{{{{\rm{local}}}}}}}=\frac{{{{{{{\rm{f}}}}}}}_{{{{{{\rm{s}}}}}}}}{{{{{{{\rm{f}}}}}}}_{{{{{{\rm{a}}}}}}}}\Delta {{{{{{\rm{T}}}}}}}_{{{{{{\rm{s}}}}}}}^{{{{{{\rm{local}}}}}}}$$

Using Eqs. (8), (11), (13), and (14), changes of air temperature change can be decomposed as:

15 $${\triangle {{{{{\rm{T}}}}}}}_{{{{{{\rm{a}}}}}}}=\frac{1}{{{{{{{\rm{f}}}}}}}_{{{{{{\rm{a}}}}}}}}\left[-{{{{{{\rm{SW}}}}}}}_{{{{{{\rm{d}}}}}}{{{{{\rm{n}}}}}}}\Delta {{\alpha }}+\left(1-{{\alpha }}\right)\Delta {{{{{{\rm{SW}}}}}}}_{{{{{{\rm{dn}}}}}}}-\Delta {{{{{\rm{LE}}}}}}-\Delta {{{{{\rm{G}}}}}}+\frac{{{\rho }}\cdot {{{{{{\rm{C}}}}}}}_{{{{{{\rm{p}}}}}}}}{{{{{{{\rm{r}}}}}}}_{{{{{{\rm{a}}}}}}}^{2}}\left({{{{{\rm{T}}}}}}_{{{{{\rm{s}}}}}}-{{{{{{\rm{T}}}}}}}_{{{{{{\rm{a}}}}}}}\right)\Delta {{{{{{\rm{r}}}}}}}_{{{{{{\rm{a}}}}}}}+{{{\varepsilon }}}_{{{{{{\rm{s}}}}}}}{{\sigma }}{{{{{{\rm{T}}}}}}}_{{{{{{\rm{a}}}}}}}^{4}\Delta {{{\varepsilon }}}_{{{{{{\rm{a}}}}}}}\right]+\Delta {{{{{{\rm{T}}}}}}}_{{{{{{\rm{a}}}}}}}^{{{{{{\rm{cir}}}}}}}$$

where $-{{{{{{\rm{SW}}}}}}}_{{{{{{\rm{d}}}}}}{{{{{\rm{n}}}}}}}\Delta {{\alpha }}$, $\left(1-{{\alpha }}\right)\Delta {{{{{{\rm{SW}}}}}}}_{{{{{{\rm{dn}}}}}}}$, $-\Delta {{{{{\rm{LE}}}}}}$, $-\Delta {{{{{\rm{G}}}}}}$, $\frac{{{\rho }}\cdot {{{{{{\rm{C}}}}}}}_{{{{{{\rm{p}}}}}}}}{{{{{{{\rm{r}}}}}}}_{{{{{{\rm{a}}}}}}}^{2}}\left({{{{{\rm{T}}}}}}_{{{{{\rm{s}}}}}}-{{{{{{\rm{T}}}}}}}_{{{{{{\rm{a}}}}}}}\right)\Delta {{{{{{\rm{r}}}}}}}_{{{{{{\rm{a}}}}}}}$, and ${{{\varepsilon }}}_{{{{{{\rm{s}}}}}}}{{\sigma }}{{{{{{\rm{T}}}}}}}_{{{{{{\rm{a}}}}}}}^{4}\Delta {{{\varepsilon }}}_{{{{{{\rm{a}}}}}}}$ represent air temperature changes induced by changes in albedo, shortwave downward radiation, latent heat flux (mainly evapotranspiration), ground heat flux, aerodynamic resistance, and air emissivity, respectively. Similar to Eq. (13), these six terms constitute the effects of the altered local surface energy balance:

16 $$\Delta {{{{{{\rm{T}}}}}}}_{{{{{{\rm{a}}}}}}}^{{{{{{\rm{local}}}}}}}=\frac{1}{{{{{{{\rm{f}}}}}}}_{{{{{{\rm{a}}}}}}}}\left[-{{{{{{\rm{SW}}}}}}}_{{{{{{\rm{d}}}}}}{{{{{\rm{n}}}}}}}\Delta {{\alpha }}+\left(1-{{\alpha }}\right)\Delta {{{{{{\rm{SW}}}}}}}_{{{{{{\rm{dn}}}}}}}-\Delta {{{{{\rm{LE}}}}}}-\Delta {{{{{\rm{G}}}}}}+\frac{{{\rho }}\cdot {{{{{{\rm{C}}}}}}}_{{{{{{\rm{p}}}}}}}}{{{{{{{\rm{r}}}}}}}_{{{{{{\rm{a}}}}}}}^{2}}\left({{{{{\rm{T}}}}}}_{{{{{\rm{s}}}}}}-{{{{{{\rm{T}}}}}}}_{{{{{{\rm{a}}}}}}}\right)\Delta {{{{{{\rm{r}}}}}}}_{{{{{{\rm{a}}}}}}}+{{{\varepsilon }}}_{{{{{{\rm{s}}}}}}}{{\sigma }}{{{{{{\rm{T}}}}}}}_{{{{{{\rm{a}}}}}}}^{4}\Delta {{{\varepsilon }}}_{{{{{{\rm{a}}}}}}}\right]$$

Temperature change induced by altered atmospheric circulation ($\Delta {{{{{{\rm{T}}}}}}}_{{{{{{\rm{a}}}}}}}^{{{{{{\rm{cir}}}}}}}$) is calculated as the residual from Eq. (11). The altered atmospheric circulation would affect air temperature far away from the regions with land cover change (e.g., the BECCS regions in this study). The relative contribution of $\Delta {{{{{{\rm{T}}}}}}}_{{{{{{\rm{a}}}}}}}^{{{{{{\rm{cir}}}}}}}$ to ΔT_a_ deduced in our study is comparable to previous estimates^23,35^ (see Supplementary Methods 2.4).

## Supplementary information

Supplementary Information

Supplementary Table 2

Supplementary Table 3

## Data Availability

The data which support the main findings of this study are provided as the Source data files of this paper, which is also available via 10.5281/zenodo.5712916. Source data are provided with this paper.

## Source data

Source Data

## Code availability

The source code of ORCHIDEE-MICT-BIOENERGY is available at 10.14768/02v2-z742, and more details can be found at https://forge.ipsl.jussieu.fr/orchidee/wiki/GroupActivities/CodeAvalaibilityPublication/ORCHIDEE_MICT_BIOENERGY_r7298. ORCHIDEE-MICT-BIOENERGY is governed by the CeCILL licence under French law and abides by the rules of the distribution of free software. One can use, modify, and/or redistribute the software under the terms of the CeCILL licence as circulated by CEA, CNRS, and INRIA at the following URL: http://www.cecill.info (last access: December 4, 2021).
