# Supplementary material for: Global cooling induced by biophysical effects of bioenergy crop cultivation
Source: Nat Commun. 2021 Dec 13;12:7255. doi: 10.1038/s41467-021-27520-0 (PMC8668960; doi:10.1038/s41467-021-27520-0)
Supplement: Supplementary file 1 — Supplementary Information [file 41467_2021_27520_MOESM1_ESM.pdf]

Global cooling induced by biophysical effects of bioenergy crop cultivation

Supplementary Information

Supplementary Methods 1. Brief introduction to the coupled model

The coupled Earth system model IPSL-CM (version 6.0.10, a modified version of IPSL-CM6A-LR)[1] was used to simulate the biophysical feedbacks of bioenergy crop cultivation. The land-surface model (ORCHIDEE)[2] and the atmosphere model (LMDZ, version 6)[3,4] serve as two components of IPSL-CM, and they were coupled through exchange of information at the interface. Other components of IPSL-CM (e.g., ocean and sea-ice models) were not activated during our simulations, because we mainly focus on the air temperature change from the perspective of the land energy budget. We therefore prescribed the sea surface temperature and sea ice to isolate energy changes over the land surface. Seasonal cycles, with no interannual variations, of sea surface temperature and sea ice were prescribed with climatological data from the Atmospheric Model Intercomparison Project (AMIP; www.pcmdi.llnl.gov/projects/amip).

We used ORCHIDEE-MICT-BIOENERGY[5] to replace the old ORCHIDEE version in the coupled model. ORCHIDEE-MICT-BIOENERGY has been developed specifically to represent bioenergy crops (see Methods)[5]. The atmosphere model used here (LMDz) includes the fundamental dynamical and physical processes of the atmosphere[3,4], and has a timestep of 2.15 min. The timestep of the land-surface model is 30 min. The spatial resolution of the coupled model is 1.26° latitude × 2.5° longitude.

Supplementary Methods 2. Validation of the IPSL-CM model

Supplementary Methods 2.1. Validation in previous studies

The IPSL-CM we used is coupled by ORCHIDEE-MICT-BIOENERGY (the land

surface model) and LMDz (the atmosphere model). Parameters related to vegetation growth for the plant functional types (PFTs) of four bioenergy crop types in ORCHIDEE-MICT-BIOENERGY have been systematically calibrated in Li et al.[5] using plant measurements, and the yields were also evaluated against a global observation-based yield dataset for major lignocellulosic bioenergy crops[6]. Fundamental dynamical and physical processes of the atmosphere simulated by LMDz have been validated by Li et al.[7] and Zeng et al.[8] against stable-isotope-based transpiration observations[8], satellite-based surface radiation data and surface energy fluxes from reanalysis data[7]. For the vegetation types other than bioenergy crops, the coupled IPSL-CM can reproduce the sensitivity of evapotranspiration to LAI changes[8] and reasonably simulate the temporal variations of surface energy fluxes[7].

In the bioenergy crop simulations in our study, we covered 3.8%±0.5% of the global total land area with bioenergy crops. In the reference simulation for the composite cultivation map, food crop was cultivated in the BECCS regions. Therefore, we further evaluated the performance of the coupled model against the observed albedo and evapotranspiration for food crops and bioenergy crops in this study.

Supplementary Methods 2.2. Validation for food crops using FLUXNET data

Observations made over food crops at 19 sites were retrieved from the FLUXNET database[9] (Supplementary Table 1) and compared with the simulated results in the corresponding grid cell with the same vegetation type. The monthly simulated albedo and evapotranspiration agree with the observed data for food crops (Supplementary Figure 1-4). The simulated albedo of food crops also captures the observed seasonal variations with significant temporal correlations ($p<0.05$) in 10 out of 12 sites (Supplementary Figure 1). In fact, 65% of the monthly albedo observations can be reproduced by the model (i.e., dots with error bars crossing the 1:1 line, Supplementary Figure 2). Similarly, the seasonal variation of observed evapotranspiration is captured by the model simulation (Supplementary Figure 3). Significant correlations between

simulated and observed evapotranspiration were found (see p values in Supplementary Figure 3 and 68% dots with error bars crossing the 1:1 line in Supplementary Figure 4.

Supplementary Methods 2.3. Validation for bioenergy crops using collated observations

Field measurements of evapotranspiration and albedo for different bioenergy crops were collated and used to evaluate the model performance. We extracted 241 observations of evapotranspiration from 77 articles and 49 observations of albedo from 28 articles. Other information (e.g., crop type, measurement time) was also recorded. After aggregating the site observations into grid cells at the model resolution ($1.26°$ latitude $\times 2.5°$ longitude), 109 observations of evapotranspiration and 36 observations of albedo were derived (Supplementary Table 2 and Supplementary Figure 5). Note that there might be several observations for one grid cell referring to different time spans (e.g., one reported averaged albedo during Jan-Mar and the other for July-Sep). We treated each of these as one individual observation to compare with the model results during the corresponding time period.

Simulated evapotranspiration (Supplementary Figure 6) and albedo (Supplementary Figure 7) generally agree with the observations, with most of the model-observation results lying around the 1:1 line. For evapotranspiration, there are 90 out of 109 points (83%) with error bars (representing the range) crossing the 1:1 line (Supplementary Figure 6), indicating that the model can at least capture some of the observations. For the other 19 points with inconsistent simulated and observed results, we listed the possible reasons for each site in Supplementary Table 3. Some main reasons include 1) the local climate different from the mean value of the whole grid cell in the model, and 2) irrigation at the observation site not represented in the model. There are also some sites in our dataset with consecutive monthly observations of evapotranspiration (Supplementary Figure 8), and the model can generally reproduce the seasonal variations of observed evapotranspiration. However, the Tarim site (purple lines in Supplementary Figure 8), located in the desert, is an exception. The climate at Tarim is

warm and dry, and site-level observations of the vegetation state may be unrepresentative of the 1.26 °(latitude) × 2.5 °(longitude) grid cell as a whole. On the other hand, the model simulation also simplified the strong heterogeneity in the grid cell, leading to a mismatch between the grid-level simulation and field observation at this particular site.

Similarly, simulated albedo is consistent with observations (Supplementary Figure 7), with 28 out of the 36 observations with error bars (the full range) crossing the 1:1 line (Supplementary Figure 7). In addition, we compared satellite-based albedo observations with the model results. Cai et al.[10] reported MODIS-based albedo for miscanthus and switchgrass in six agro-ecological zones (AEZ7-AEZ12) in the US. We extracted the simulated albedo in the region studied by Cai et al.[10] and compared these values with the observations. The simulated albedo throughout the year generally agrees with the MODIS-based albedo values from Cai et al.[10] both for miscanthus and switchgrass (Supplementary Figure 9).

Supplementary Methods 2.4. Comparison of the relative contribution of ΔT_a^{cir} to ΔT_a with previous studies

We also compared the relative contribution of ΔT_a^{cir} to ΔT_a deduced in our study with previous estimates[8,11]. ΔT_a^{cir} is close to ΔT_a from Luyssaert et al.[11] for a future forest management scenario in European forest, because the components of ΔT_a^{local} offset each other, resulting in a value of ΔT_a^{local} of rather small magnitude (Figure 2b in Luyssaert et al.[11]). Zeng et al.[8], found that ΔT_a^{cir} contributes over 40% to global ΔT_a induced by increased LAI (comparing Figure 1a and Supplementary Figure 3b in Zeng et al.[8]). In our study, ΔT_a^{cir} contributes 21%-79% to global ΔT_a for the four bioenergy crop scenarios (Figure 2a).

Supplementary Methods 2.5. Comparison of the biophysical effects with previous study

We also compare the biophysical effects on air temperature (ΔT_a) in our study against precious study over the central US[12]. Georgescu et al.[12] simulated the biophysical

effects of miscanthus (84 M ha) and maize in the central US by modifying the surface vegetation parameters of the Weather Research and Forecasting (WRF) model based on observations. They found that compared to maize, miscanthus cultivation has cooling effects (ΔT_a = -0.45 ~ -0.84 °C in the perturbed pixels, and = -0.07 ~ -0.16 °C in all land pixels of the contiguous US, northern Mexico and southern Canada), mainly because of the enhanced evapotranspiration and decreased net surface shortwave and longwave radiation. Compared to Georgescu et al.[12], our results for miscanthus cultivation in the central US (41 M ha) also show reduced air temperature (ΔT_a = -0.27 °C in the cultivation regions, and = -0.12 °C in the contiguous US), mainly contributed by higher aerodynamic resistance (which was omitted in Georgescu et al.[12]), enhanced evapotranspiration and increased albedo.

Supplementary Methods 3. Bioenergy crop cultivation distribution

Supplementary Methods 3.1. Bioenergy crop cultivation maps

Large-scale cultivation of lignocellulosic bioenergy crops would inevitably compete for land against the original land use types (e.g., agricultural land or forest). To minimize this land competition, bioenergy crops are recommended to be planted on "marginal land"[13]. Marginal land mainly refers to agricultural land abandoned due to degradation, low profitability or environmental and ecological conservation[14,15]. To generate the bioenergy crop cultivation map (i.e., the BECCS regions used in this study), we combined global marginal land datasets from Campbell et al.[16] and Cai et al.[17] and bioenergy cultivation maps from future BECCS scenarios of two IAMs (MAgPIE[18] and IMAGE[19]).

Marginal land, as assessed by Campbell et al.[16], is based on historical land-use changes (History Database of the Global Environment, HYDE 3.0[20]) and land-cover maps derived from MODIS. There are two maps with different assumptions of land-use transitions: Scenario High and Scenario Low (Campbel-high and Campbell-low, hereafter). In Campbell-high, the largest cropland area in each grid cell since 1700 was

compared with the cropland area in 2000 and the difference was regarded as the abandoned cropland area in that grid cell. The abandoned pasture area was calculated in the same way. In Campbell-low, conversion from pasture to cropland and conversion from cropland to pasture were considered. Therefore, only the decrement of total area of both cropland and pasture land was recognized as marginal land. In both scenarios from Campbell et al.[16], transition from agricultural land to urban or forest was excluded from the marginal land area.

Cai et al.[17] first estimated land productivity using land properties like soil productivity, land slope, soil temperature and humidity and then classified the marginal land based on the land productivity: low, marginal and regular productivity. There are four scenarios of marginal land from Cai et al.[17]. Scenario 1 (used in this study) considers only land with mixed crops and natural vegetation with marginal productivity. Scenario 2 adds marginal cropland on top of scenario 1; scenario 3 further adds marginal grassland, savanna and shrubland on to scenario 2; scenario 4 removes the pasture land from scenario 3.

The BECCS scenarios from MAgPIE[18] and IMAGE[19] were developed based on Representative Concentration Pathway (RCP) 2.6 and Shared Socio-economic Pathway (SSP) 2, where BECCS serves as the only negative emission technology option. Both IAMs harmonized the historical patterns of cropland and pasture land according to the HYDE 3.1 dataset[21]. In MAgPIE, competition between bioenergy crop cultivation and cropland is allowed[18], while bioenergy crop cultivation must evade the food-production lands in IMAGE[19].

The global total area available for bioenergy crop cultivation is 459.5 and 365.6 M ha for Campbell-high and Campbell-low, 425.6, 948.5, 1776.3 and 1418.9 M ha, for scenarios 1 to 4, respectively, of Cai et al.[17], 523.4 M ha in 2100 for IMAGE and 407.7 M ha in 2100 for MAgPIE, calculated from the 0.5° resolution maps. The areas of land available for bioenergy crop cultivation in scenarios 2 to 4 of Cai et al.[17] (1381.2 M ha

on average) are significantly higher than for other datasets (436.4 M ha on average) and may be unrealistic compared to that in IAMs. Therefore, we only used cultivation maps datasets from Campbell-high, Campbell-low, Scenario 1 of Cai et al.[17] (Cai S1, hereafter), IMAGE and MAgPIE (Supplementary Figure 10) to generate an idealized composite map for bioenergy crop cultivation.

Supplementary Methods 3.2. Composite bioenergy crop cultivation map

One reason for using a composite map is to test the different biophysical effects of the four bioenergy crop types, and one composite map containing the cultivation information from all five maps would help save the computational resources. As for the sensitivity of biophysical effects to various cultivation maps, we performed additional simulations using three individual cultivation maps and two representative bioenergy crops (Supplementary Methods 4). Another reason is that because some biophysical variables in the model are not PFT specific (i.e., one value over the whole grid cell), if we used fractional PFT coverage in the grid cells with bioenergy cultivation, the biophysical signals are thus a mixture of all PFTs in these grid cells. As a result, we cannot separate the impacts in the BECCS region and outside the BECCS region (Figure 2). The composite cultivation map for idealized simulations was generated by the following steps:

1) We converted these five map**s** (Supplementary Figure 10) to the resolution of the coupled model (1.26° latitude × 2.5° longitude). For each dataset, we calculated the land area available for bioenergy crop cultivation in each grid cell at the new resolution using the total land area in the grid cell multiplied by the land fraction for bioenergy crop cultivation.

2) We calculated the land area available for bioenergy crop cultivation averaged over the five datasets in each grid cell (1.26° latitude × 2.5° longitude).

3) We arranged all land grid cells globally in a descending order of the mean bioenergy crop cultivation area calculated in the last step.

4) We selected the grid cells with the highest land area available as the most likely grid cells for bioenergy cultivation and covered the whole grid cell with bioenergy crops until the total area of the selected grid cells reaching 465.6 M ha (the mean value of IMAGE and MAgPIE, which were developed based on RCP 2.6 and SSP2).

The selected grid cells in the composite cultivation map were thus used as bioenergy crop cultivation regions (i.e., BECCS regions in this study) to drive the coupled model (Figure 1a). For the idealized simulations, the corresponding bioenergy crops were cultivated in the BECCS regions according to the composite map. Because the original source vegetation in the BECCS regions was mainly short vegetation (Supplementary Figure 11), and we assumed that bioenergy crop should be conservatively cultivated only on lands with short vegetation to get rid of deforestation, the BECCS source regions were covered by the generic food crop PFT in the reference simulation (S_{ref}, see details in Supplementary Methods 4). The BECCS regions in the composite map are distributed from 38°S to 60°N (Figure 1a).

Supplementary Methods 4. Simulating biophysical effects of bioenergy crop cultivation

Supplementary Methods 4.1. Simulations based on the composite cultivation map

Large-scale bioenergy crop cultivation changes air temperature mainly through: 1) altering the atmospheric CO_2 concentration (e.g., CO_2 removal by vegetation growth and CO_2 emission from land cover changes), which modifies the greenhouse effect; and 2) altering global energy cycling (e.g., changed surface albedo and disturbed evapotranspiration), which changes temperature through biophysical processes. Our study focused on the latter, specifically the biophysical effect on temperature of bioenergy crop cultivation, and targeted it with the following scenarios based on the composite cultivation map:

1) A reference scenario (S_{ref}), with BECCS regions in the composite cultivation map covered by generic food crop vegetation. This scenario was initially run for 50 years

with constant atmospheric CO_2 concentration, unchanged land cover (that of 2015) and with sea surface temperature and sea ice extent prescribed by climatology data. We used the generic grain crop vegetation as the reference scenario because it was proposed to deploy bioenergy crops on marginal land (mainly abandoned agricultural land with short vegetation, Supplementary Figure 11) to avoid deforestation and direct land competition with food crops.

2) Four idealized bioenergy crop scenarios, in which the BECCS regions in the composite cultivation map were covered by one of the four individual bioenergy crop types (i.e., S_{euc}: eucalypt, $S_{p\&w}$: poplar & willow, S_{mis}: miscanthus, S_{swi}: switchgrass), were conducted to simulate the biophysical effects of large-scale bioenergy crop cultivation. All the other settings of these scenarios are the same as in S_{ref}.

3) We analyzed the multi-year mean values of outputs from all scenarios in order to obtain relatively stable results globally. To avoid the perturbations caused by the regular harvest of woody bioenergy crop cultivation (i.e., every 5 years), we averaged the outputs over the last 10 years of the simulations. The differences between the bioenergy crop scenarios and the reference scenario were assumed to be the changes induced by cultivation of each bioenergy crop.

The simulations were run for 50 years, which covers ten regular rotation cycles of woody bioenergy crops periods (harvested in every five years). In these simulations, the key vegetation features like leaf area index (LAI) and gross primary productivity (GPP) that are closely associated with the energy balances (e.g., evapotranspiration (ET) and albedo) generally reach a steady state after 5~10 years of the cultivation for all bioenergy crop types (Supplementary Figure 12). In addition, we averaged the results over the 41st – 50th year, the 36th – 50th year and the 31st – 50th year of the simulations to test the robustness against different timespans of aggregation (Supplementary Figure 13). The results averaged over the 31st – 50th year or the 36th – 50th year of the

simulations generally agree with those over the last ten years (i.e., 41st – 50th year, used as the main results in this study). Therefore, the 50-year simulations are sufficient to derive a robust signal of the biophysical effects from bioenergy crop cultivation, and our main results using the data from the last ten years are also robust regardless of the choices in various analyzing periods.

Supplementary Methods 4.2. Additional simulations for sensitivity tests

In addition to the four idealized simulations based on the composite cultivation map (S_{euc}, $S_{p\&w}$, S_{mis} and S_{swi}), we made another six simulations based on more realistic individual cultivation maps to test the sensitivity of biophysical effects to different maps (Supplementary Table 4). Because the coupled simulations are very computational-resource consuming, in the additional runs, we selected three representative maps out of the five maps (Supplementary Figure 10a-e): 1) global marginal land map from Campbell et al.[16] (Campbell-high) with abandoned agricultural lands as the cultivation area, which is widely distributing across the globe and covered by short vegetation, 2) the bioenergy crop cultivation map from IMAGE with cultivation area converted mainly from forest and very few from croplands, avoiding competing for lands with food crops, and 3) the bioenergy crop cultivation map from MAgPIE with the cultivation lands mainly converted from croplands because MAgPIE allows land competition between bioenergy crops and food crops based on cost minimization (Supplementary Table 4, Supplementary Figure 10). Cultivation maps from the two IAM scenarios considered BECCS as the only land-based negative emission option in limiting global warming in the future, and these models didn't limit bioenergy crop cultivation on marginal lands. These three selected maps are representative because they cover various total cultivation areas, different spatial distribution patterns and different land sources. Global total area for bioenergy crop cultivation in these three selected maps ranges from 408 to 523 M ha (88% to 112% of the BECCS area in the composite map, Supplementary Table 4, Supplementary Figure 10). Compared to the cultivation map from Campbell-high, bioenergy crop cultivation area from MAgPIE and IMAGE mainly concentrated in a few regions (e.g., Europe, central North America and central Africa). The bioenergy crop cultivation lands from IMAGE are mostly (78%) converted from forest, while cultivation lands from MAgPIE and Campbell-high are mainly from short vegetation (Supplementary Figure 10). For the bioenergy crop types,

we used eucalypt and switchgrass, representing one woody and one herbaceous crop, due to their contrasted biophysical effects in the simulations of the composite map (Figure 1 and 2). In total, six additional experimental simulations (3 individual cultivation maps × 2 bioenergy crop types) were run (i.e., S_{euc}^{MAg}, S_{euc}^{IMA}, S_{euc}^{Cam}, S_{swi}^{MAg}, S_{swi}^{IMA} and S_{swi}^{Cam} in Supplementary Table 4).

To match the six additional more realistic simulations, we also run a corresponding reference simulation (S_{ref}^{pre}) with present-day land cover map in 2014[22]. Note that S_{ref}^{pre} of the six additional more realistic simulations is different from S_{ref} of the four idealized simulations (S_{euc}, $S_{p\&w}$, S_{mis}, S_{swi}) using the composite map. The idealized simulations are based on the composite map assuming that the whole grid cells in the cultivation regions were converted to bioenergy crops from marginal lands, originally with short vegetation. Therefore, generic food crops were cultivated in the BECCS regions to represent short vegetation in the S_{ref}, not the present land covers as in S_{ref}^{pre}. However, in the three individual maps, bioenergy crop cultivation area was fractional in each grid cell (not the whole grid cells as in the composite map) converted from different land sources. The present land cover map should thus be used in the corresponding reference simulation (S_{ref}^{pre}). The biophysical effects based on the three individual cultivation maps (shown in Supplementary Table 4) are calculated by S_{ibio}^{imap} - S_{ref}^{pre}, where imap = IMAGE, MAgPIE or Campbell-high, and ibio = eucalypt or switchgrass. Except for the cultivation maps, other settings in the simulations in Supplementary Table 4 are the same as the idealized simulations based on the composite map (S_{euc}, $S_{p\&w}$, S_{mis}, S_{swi}). Differences in the changes of biophysical effects between simulations using the idealized composite map and more realistic individual maps thus result from differences in the total cultivation areas, spatial distribution patterns, land sources and bioenergy crop types.

Supplementary Methods 4.3. Sensitivity of the IAV of ΔT_a on the simulation length

The interannual variability (IAV) of global ΔT_a is relatively large compared to the magnitude of global ΔT_a averaged over the last 20 years. The global ΔT_a from the

simulation based on the composite cultivation map is 0.03 – 0.08 °C for different bioenergy crop types, while the corresponding IAV is 0.16 – 0.19 °C. The high IAV of global ΔT_a (0.16 – 0.19 °C) is mainly determined by ΔT_a^{cir} (IAV = 0.17 – 0.20 °C), which varies remarkably across different years, while ΔT_a^{local} generally shows a cooling effect with a lower IAV (0.02 – 0.04 °C), indicating the robust response of local temperature. In addition, to test the impact of simulation length on the IAV of global mean ΔT_a, we extended the eucalypt cultivation scenario based on the IMAGE map (S_{euc}^{IMA}) for another ten years (60 years in total) as an example. The IAV of ΔT_a aggregated during the extended ten years (i.e., the 51st – 60th year) of the simulation didn't show significant differences from the IAV aggregated over other timespans of the simulation (i.e., the 31st – 40th year, the 41st – 50th year and the 31st – 60th year, Supplementary Figure 20). Therefore, the IAV of global mean ΔT_a is mainly contributed by the IAV of ΔT_a^{cir}, and the simulation length seems to have little impact on the IAV.

Supplementary Discussion 1. Mechanisms leading to larger temperature change magnitudes in woody bioenergy crop scenarios

In the bioenergy crop cultivation regions based on the composite cultivation map (i.e., the BECCS regions), the magnitudes of energy flux changes in the woody bioenergy crop scenarios are greater than those in the herbaceous bioenergy crop scenarios (Figure 2b). Compared to herbaceous crops, woody crops have higher LAI (Supplementary Figure 22 and 23) and deeper roots[23] and, as a consequence, stronger evapotranspiration (Figure 2b). The high LAI of woody bioenergy crops corresponds to a lower surface albedo in the BECCS regions (Figure 2b) and thus more radiation being absorbed by the land surface[24]. Planting woody bioenergy crops may increase aerodynamic resistance through reducing wind speed, but it can also decrease aerodynamic resistance through reducing atmosphere stability and enhancing surface roughness (i.e., increasing effective surface roughness height) (Supplementary Figure 22 and 23). As a combination of these impacts, aerodynamic resistance over the BECCS regions is

generally reduced by woody bioenergy crop scenarios, leading to a cooling effect (Figure 2b). In addition, enhanced evapotranspiration from woody bioenergy crop cultivation increases air humidity and further increases cloud fraction (Supplementary Figure 22 and 23). This increased low-level cloud cover (Supplementary Figure 22 and 23) reduces the amount of downward shortwave radiation reaching the land surface through decreased air transmissivity, leading to further surface cooling (Figure 2b)[25-28]. An increase in high-level cloud cover means more longwave radiation from the surface is absorbed and mostly emitted back to the surface[29], leading to higher air emissivity for longwave radiation (warming effect, Figure 2b) in the BECCS regions[25-27]. These warming effects due to increased air emissivity and decreased albedo are counteracted by the cooling effects of enhanced evapotranspiration and reduced aerodynamic resistance, which decrease the local surface energy in the BECCS regions in the woody bioenergy crop scenarios (Figure 2b).

Supplementary Discussion 2. Difference between scenarios based on Campbell-high cultivation map and those based on other maps

The widespread BECCS cultivation in the Campbell-high map thus alters more grid cells across the globe. For example, there are 45,514 1.26° × 2.5° grid cells with bioenergy crop cultivation, which is 4.5 times of those in the IMAGE map (8,249 gird cells) and 5.5 times of those in the MAgPIE map (10,052 grid cells). Therefore, the eucalypt cultivation scenario based on the Campbell-high map brings stronger impact on both local energy budget and atmospheric circulation (i.e., larger magnitude of both ΔT_a^{local} and ΔT_a^{cir}) than those based on the other maps (Figure 4). For switchgrass cultivation, however, the temperature responses are generally gentle, with small differences of ΔT_a among various cultivation maps (Figure 4), probably due to the lower biophysical changes when replacing current vegetation with switchgrass compared to eucalypt (Figure 2b). The differences between the scenario based on the Campbell-high map and the other maps emphasize the importance of the spatial cultivation patterns on

the biophysical effects.

Supplementary Discussion 3. Differences between the two reference scenarios

When comparing the changes of biophysical effects from the composite map with those from the three individual maps, the differences in the reference map also matters. The reference simulation (S_{ref}^{pre}) for the six additional experimental simulations (3 individual cultivation maps × 2 bioenergy crop types) used the present-day map with observed fractional land covers in the bioenergy cultivation grid cells. By contrast, the simulations using the idealized composite map assumed that bioenergy crops were cultivated on the marginal lands with short vegetation, and the map in the corresponding reference simulation (S_{ref}) covers the whole bioenergy cultivation grid cells with generic food crops. Therefore, differences induced by the two reference simulations also partly reflect the impacts of different land sources (present land covers vs. cropland) for the bioenergy crop cultivation. Compared to S_{ref}^{pre}, S_{ref} generally shows higher T_a in the boreal regions and lower T_a in the pantropical regions (Supplementary Figure 33), partly contributing to the stronger cooling signals in the boreal regions and weaker cooling signals in the pantropical regions from idealized simulations using the idealized composite maps (S_{euc}, S_{swi}) than the six additional simulations using more realistic individual maps (S_{euc}^{MAg}, S_{euc}^{IMA}, S_{euc}^{Cam}, S_{swi}^{MAg}, S_{swi}^{IMA} and S_{swi}^{Cam}, Supplementary Figure 30).

Supplementary Table 1. Comparison between simulated albedo and evapotranspiration (ET, mm day^{-1}) and observations from the FLUXNET2015 database for cropland.

Site ID	*Longitude*	*Latitude*	*Observed albedo*	*Simulated albedo*	*Observed ET*	*Simulated ET*	*Begin year*	*End year*
BE-Lon	4.75	50.55	0.17	0.14	1.03	1.46	2004	2014
CH-Oe2	7.73	47.29	/	/	1.87	1.36	2004	2014
DE-Geb	10.91	51.10	0.20	0.18	0.97	1.29	2001	2014
DE-Kli	13.52	50.89	0.21	0.22	0.91	1.37	2004	2014
DE-RuS	6.45	50.87	0.14	0.16	1.75	1.36	2011	2014
DE-Seh	6.45	50.87	/	/	1.59	1.36	2007	2010
DK-Fou	9.59	56.48	/	/	0.26	0.97	2005	2005
FI-Jok	23.51	60.90	0.33	0.33	0.56	1.07	2000	2003
FR-Gri	1.95	48.84	0.17	0.15	1.36	1.51	2004	2014
IT-CA2	12.03	42.38	0.10	0.13	1.24	1.70	2011	2014
US-ARM	-97.49	36.61	0.20	0.19	1.40	2.08	2003	2012
US-CRT	-83.35	41.63	0.22	0.22	1.82	1.83	2011	2013
US-Lin	-119.84	36.36	/	/	0.93	1.24	2009	2010
US-Ne1	-96.48	41.17	0.21	0.25	1.88	1.64	2001	2013
US-Ne2	-96.47	41.16	0.22	0.25	1.89	1.64	2001	2013
US-Ne3	-96.44	41.18	0.23	0.25	1.72	1.64	2001	2013
US-Tw2	-121.64	38.10	/	/	1.69	1.58	2012	2013
US-Tw3	-121.65	38.12	/	/	2.84	1.58	2013	2014
US-Twt	-121.65	38.11	/	/	2.89	1.58	2009	2014

Supplementary Table 2. Albedo and evapotranspiration of different bioenergy crops from field measurements.

<u>See the excel file.</u>

Supplementary Table 3. Comparison between the simulated evapotranspiration and observed evapotranspiration and the possible reasons for the inconsistency of the 19 points shown as black circles in **Supplementary Figure 6**.

<u>See the excel file.</u>

Supplementary Table 4. Additional more realistic simulations based on three individual cultivation maps and the representative bioenergy crop types.

Simulation name	Cultivation data source	Global total cultivation area (M ha)	Land source for bioenergy crops globally	Bioenergy crop type
S_{euc}^{Cam}	Campbell-high	460	F=0%, C=26%, P=39%, G=35% *	Eucalypt
S_{swi}^{Cam}	Campbell-high	460	F=0%, C=26%, P=39%, G=35%	Switchgrass
S_{euc}^{IMA}	IMAGE	523	F=78%, C=2%, P=9%, G=10%	Eucalypt
S_{swi}^{IMA}	IMAGE	523	F=78%, C=2%, P=9%, G=10%	Switchgrass
S_{euc}^{MAg}	MAgPIE	408	F=5%, C=75%, P=12%, G=8%	Eucalypt
S_{swi}^{MAg}	MAgPIE	408	F=5%, C=75%, P=12%, G=8%	Switchgrass
S_{ref}^{pre}	None	0	None, using the present vegetation distribution as reference	None

* F, C, P and G represent forest, cropland, pasture and grassland, respectively. The data are the area percentage of each land cover to the total area converted to bioenergy crop cultivation lands.

Supplementary Table 5. Estimated biogeochemical cooling effect of CDR by BECCS based on transient climate response to cumulative CO_2 emissions (TCRE).

TCRE (°C·EgC^{-1})	TCRE references	Estimated biogeochemical cooling effect (°C) of CDR by BECCS from IAMs (i.e., calculated as 128 PgC×TRCE)
0.8~2.5	*MacDougal et al.*[29]*; Tokarska et al.*[31]	0.10~0.32
0.8~2.4	*CMIP5, from Gillett et al.*[32]	0.10~0.31
0.7~2	*Gillett et al.*[32]	0.09~0.26
1~2.1	*Matthews et al.*[33]	0.13~0.27

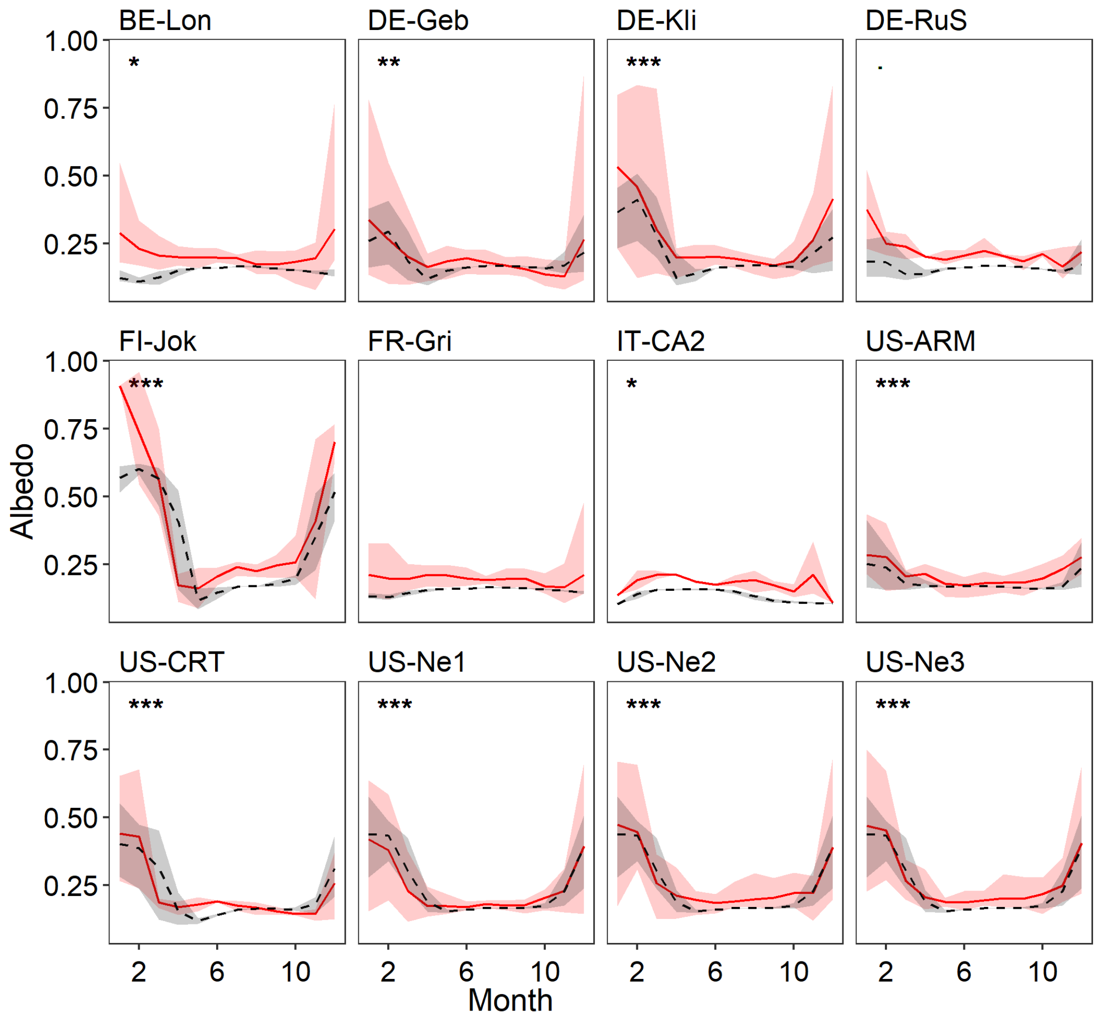

Supplementary Figure 1. Comparison between the variation of simulated albedo and observed albedo from FLUXNET sites for cropland. Black dashed lines and red lines represent simulated and observed albedo, respectively, with the shaded area representing the inter-annual range. The asterisks in each panel (and in the following figures) indicate the significance of the correlation coefficient between the simulated results and observed values. “ ”, “*”, “**”, “***” indicate the p value <0.1, < 0.05, <0.01, <0.005, respectively.

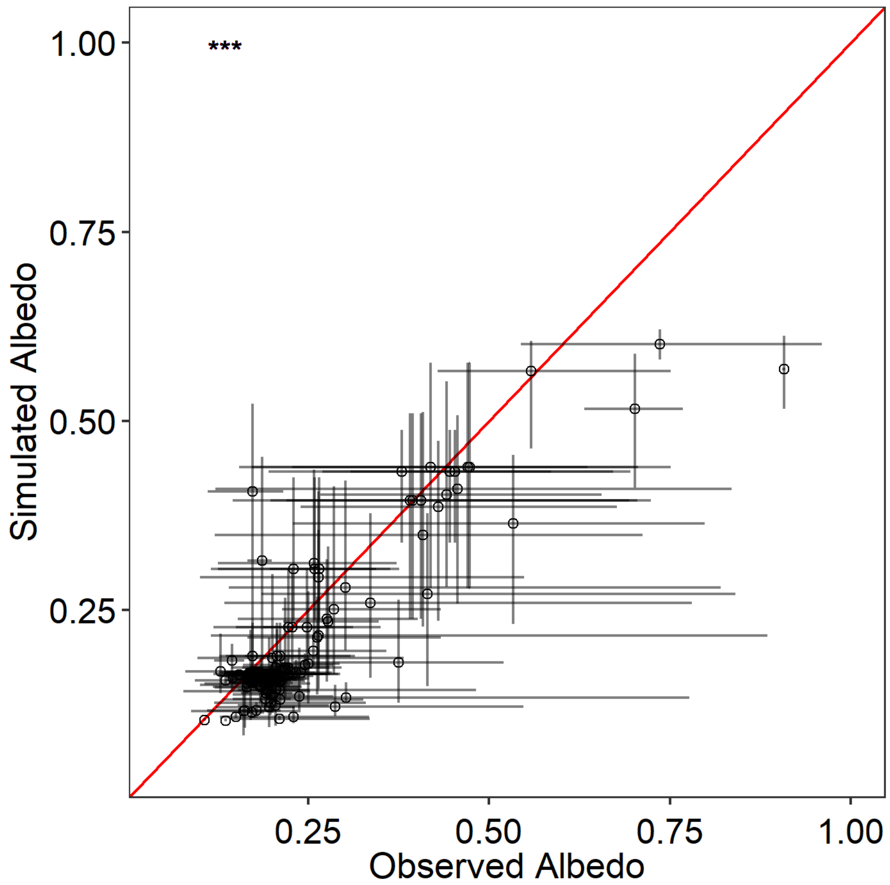

Supplementary Figure 2. Comparison between the monthly simulated albedo and observed albedo for the FLUXNET cropland sites. The error bars show the range of field observations and simulated results. The asterisks (***) indicate the significance of the correlation coefficient between the simulated results and observed values (p value < 0.005)**.**

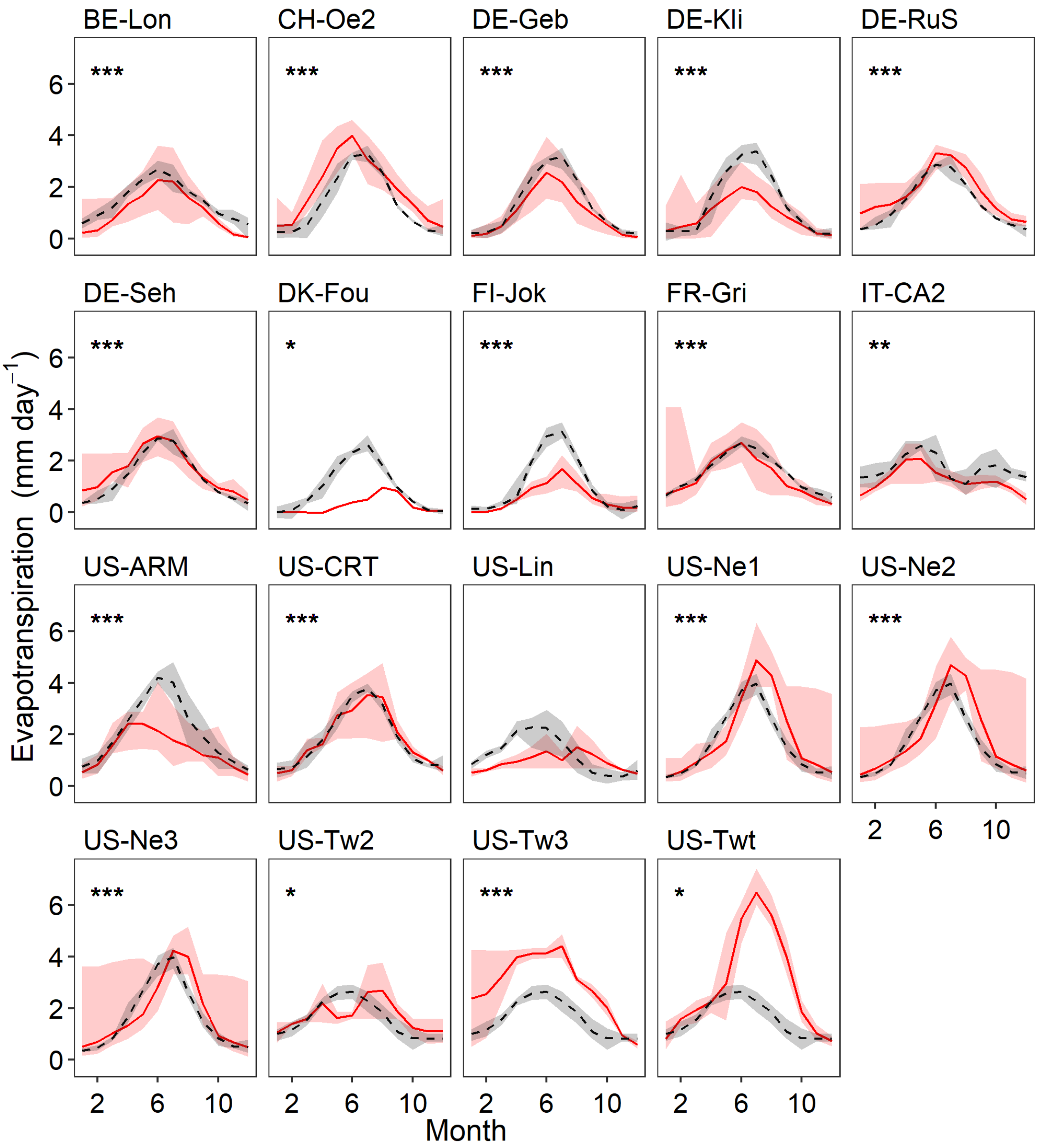

Supplementary Figure 3. Comparison between the variation of simulated evapotranspiration and observed evapotranspiration at the FLUXNET cropland sites. Black dashed lines and red lines represent simulated and observed evapotranspiration, respectively. The shaded areas represent the inter-annual range. Other notation is as described in **Supplementary Figure 1**.

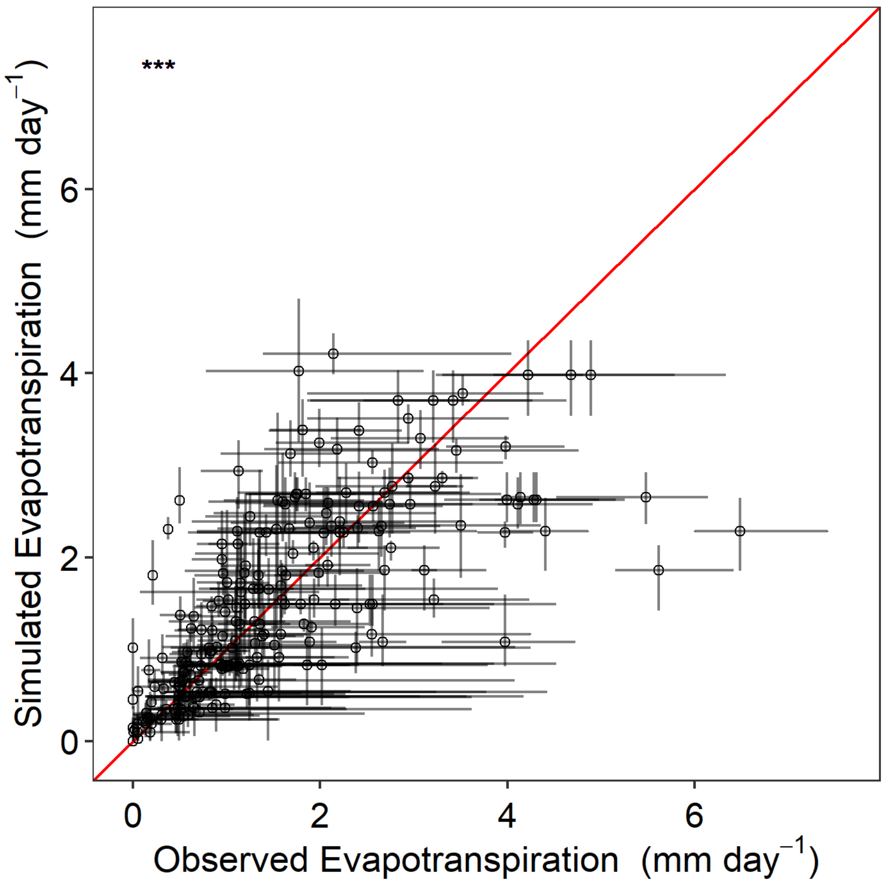

Supplementary Figure 4. Comparison between the monthly simulated evapotranspiration and observed evapotranspiration at the FLUXNET cropland sites. Other notation is as described in **Supplementary Figure 2**.

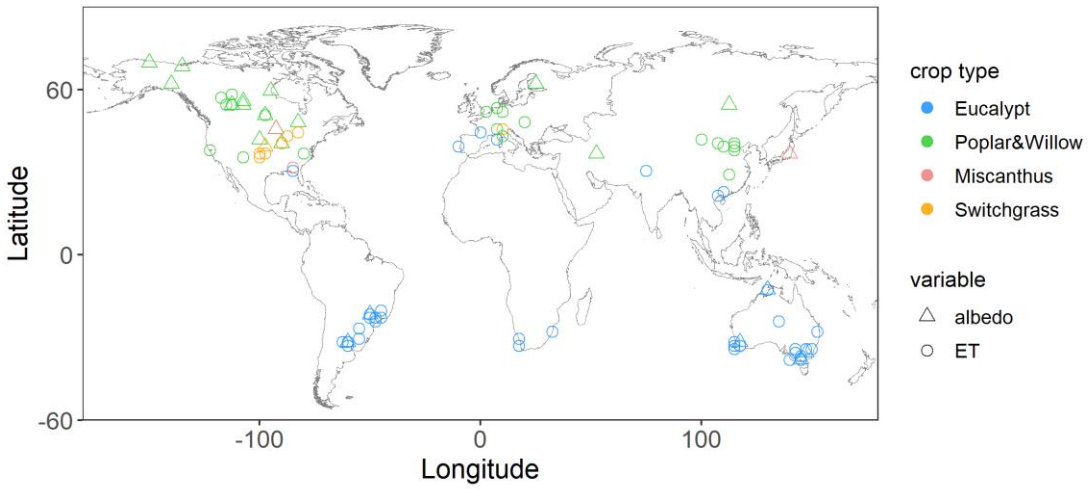

Supplementary Figure 5. Distribution of the collated observations of evapotranspiration (ET) and albedo for bioenergy crops across the 1.26 ° (latitude) × 2.5 ° (longitude) grid cells.

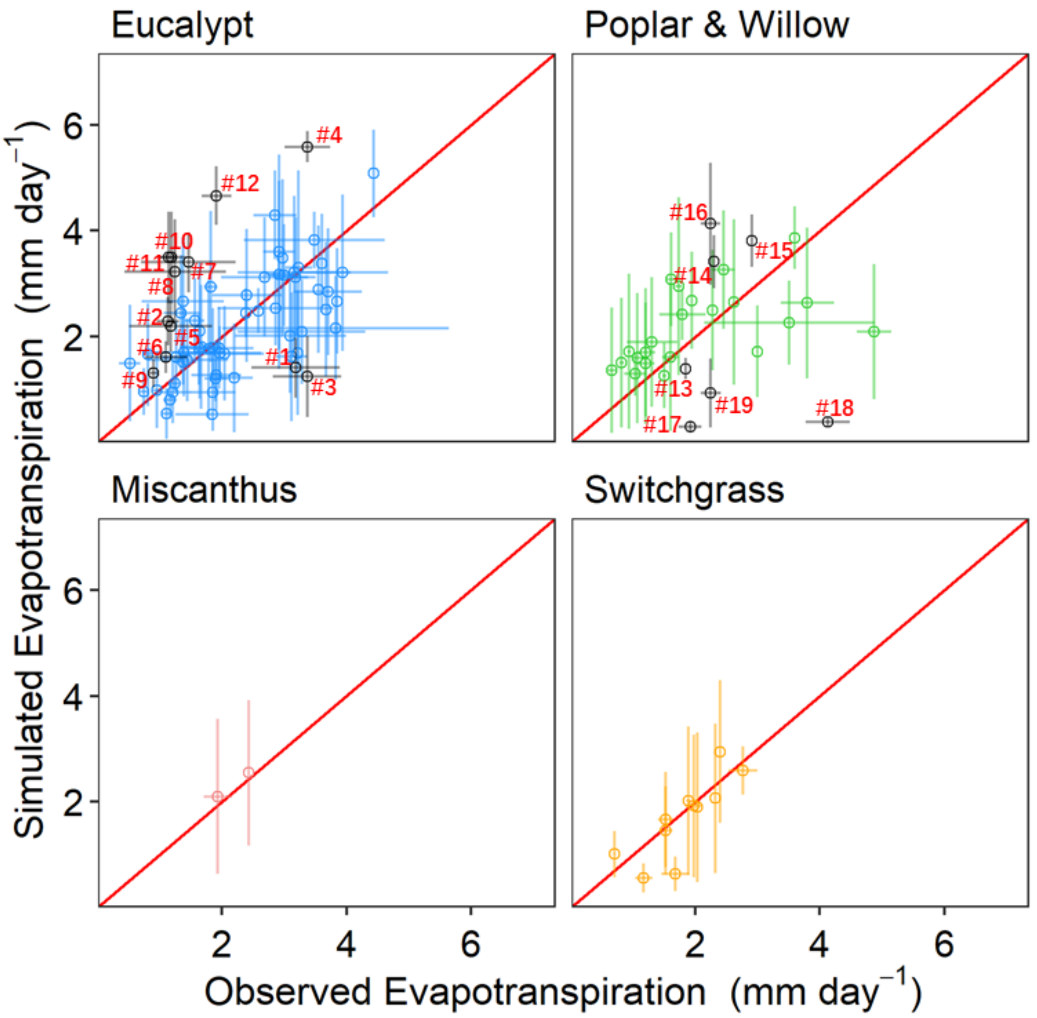

Supplementary Figure 6. Comparison between the simulated evapotranspiration and observed evapotranspiration for four bioenergy crops. The error bars show the range of field observations and model simulations. The points with mismatching results between field observations and model simulations are labelled **#1-#19** in reds (more details in **Supplementary Table 3**), shown in black circles and grey error bars.

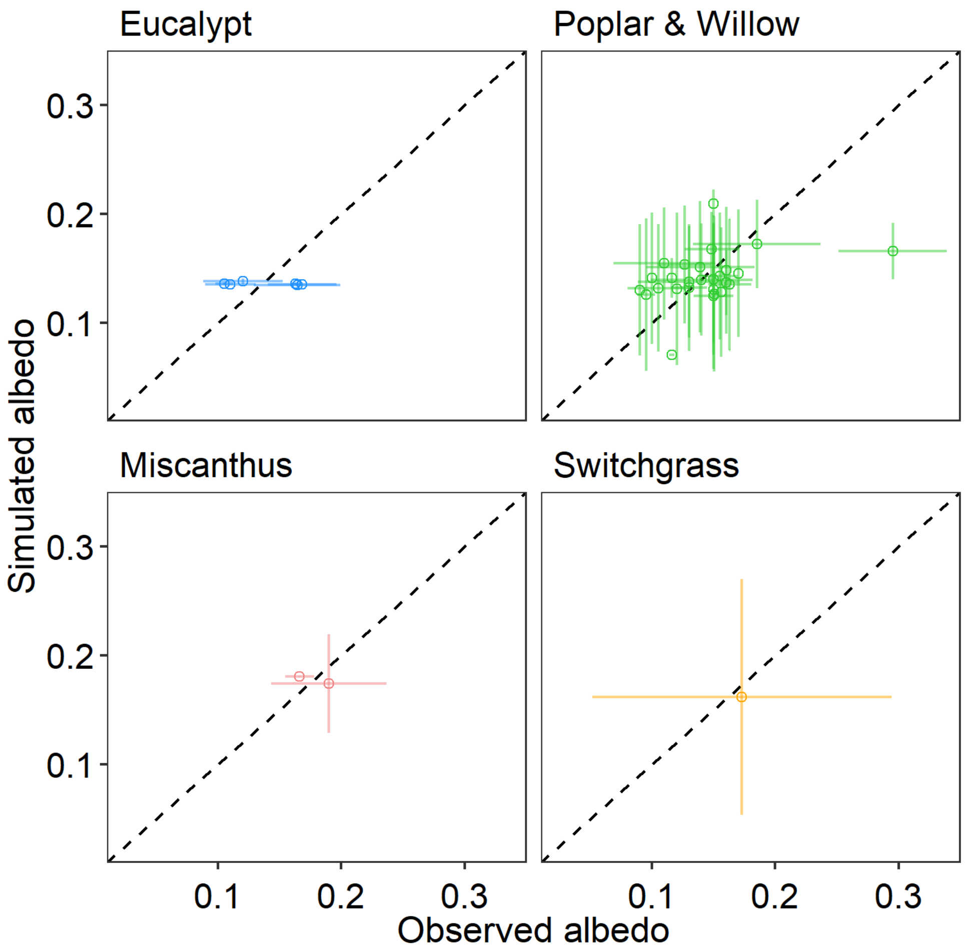

Supplementary Figure 7. Comparison between the simulated albedo and observed albedo for four bioenergy crops. The error bars show the range of field observations and model simulations.

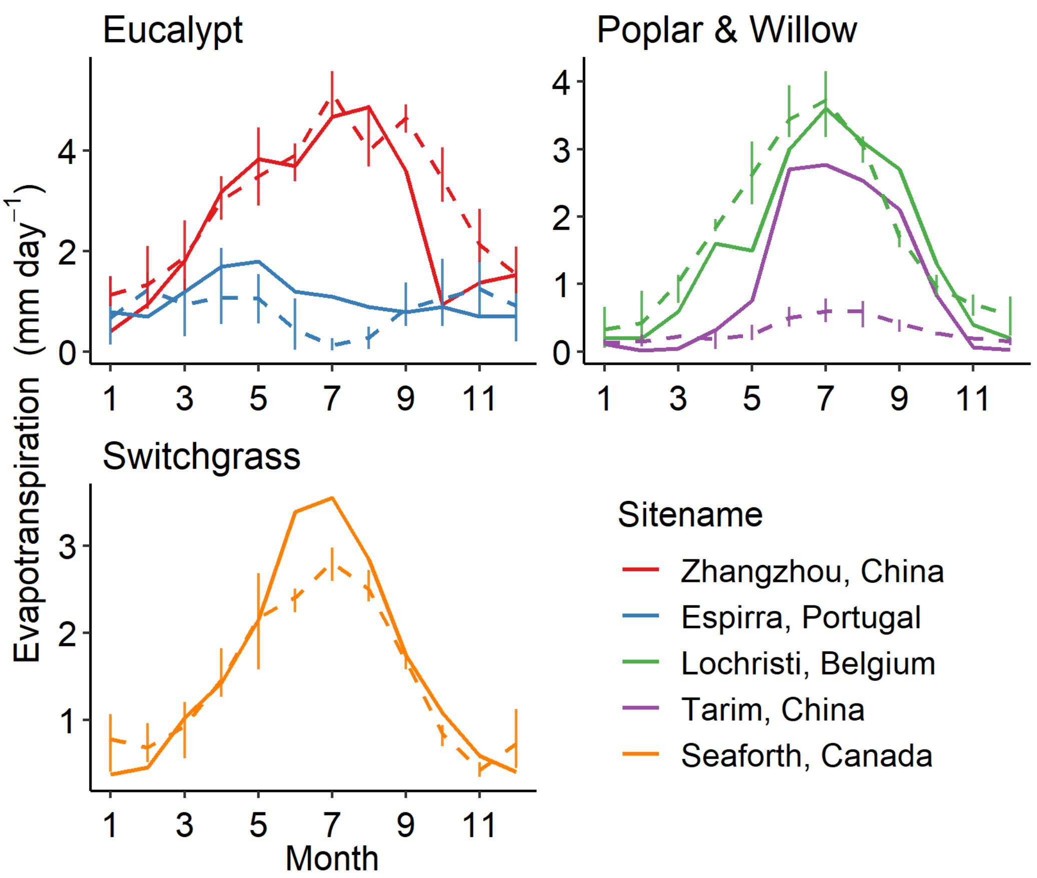

Supplementary Figure 8. Variation of observed evapotranspiration (solid lines) and simulated evapotranspiration (dashed lines) at five sites for different bioenergy crops.

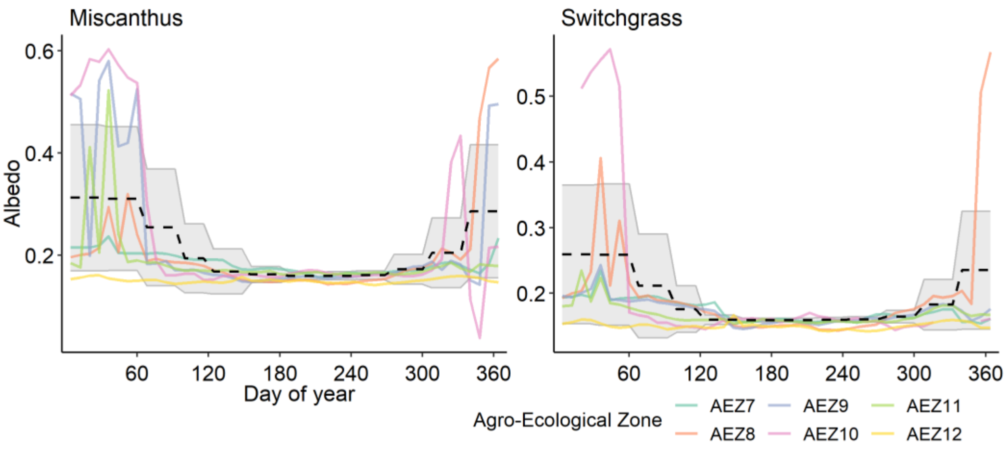

Supplementary Figure 9. Variation of MODIS albedo in six agro-ecological zones of the US mainland from Cai et al.[17] (colored lines) and the simulated albedo (black dashed line, with grey shading representing the spatial range) within the study region of Cai et al.[17].

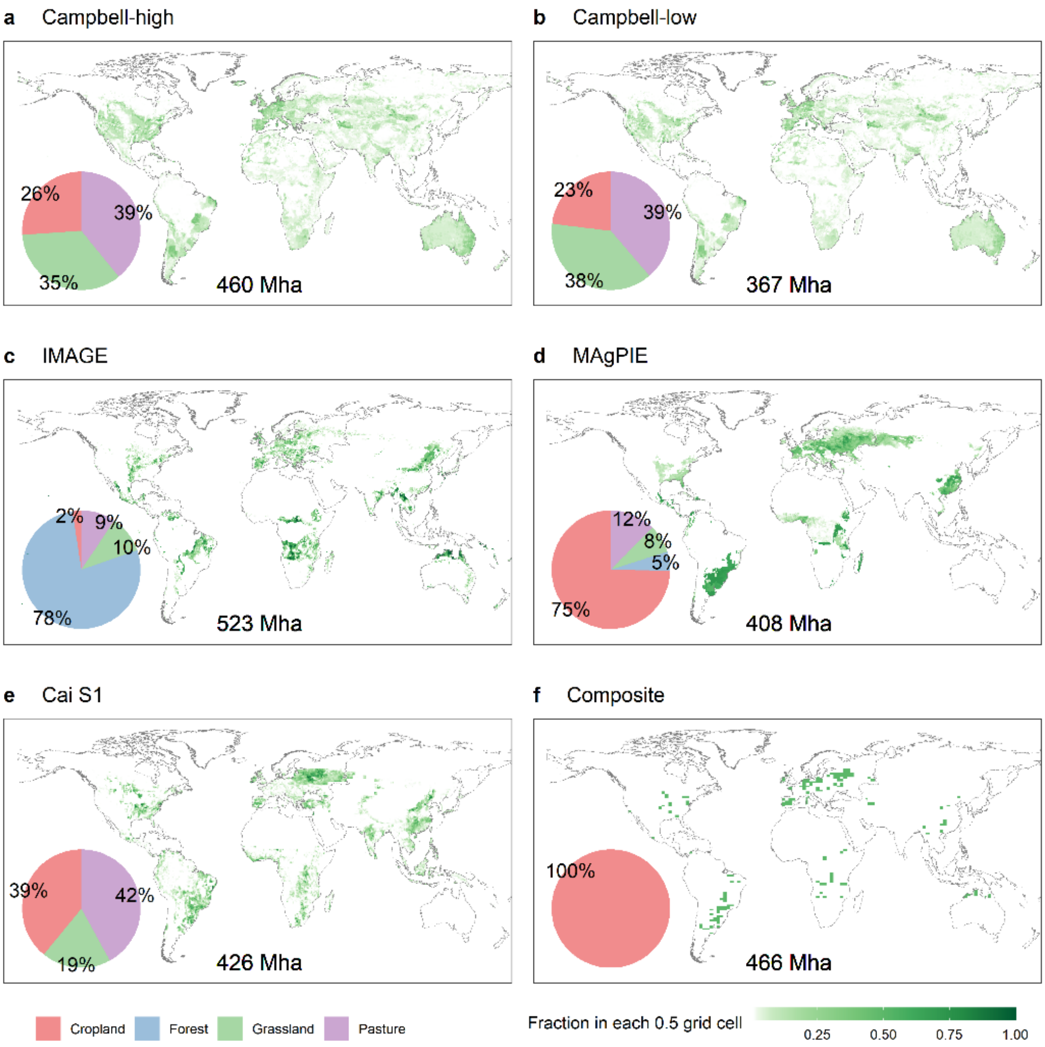

Supplementary Figure 10. Bioenergy crop cultivation lands from the five maps and the composite map. The pie chart in each panel is the area percentage of source land cover types converted to bioenergy crop cultivation lands. Global total cultivation area for each map is also shown.

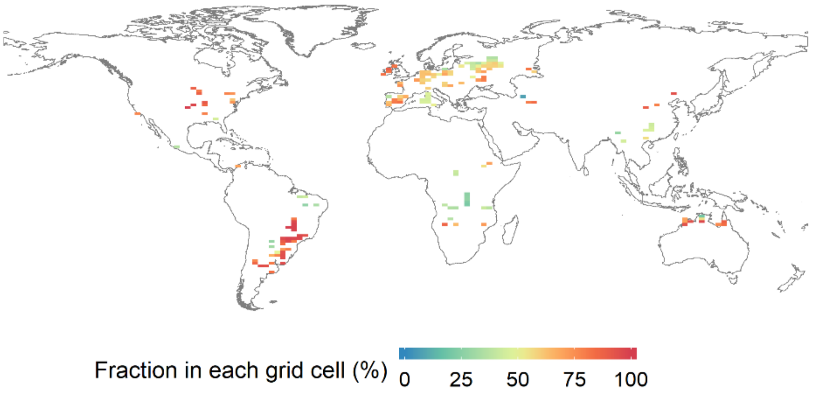

Supplementary Figure 11. The fraction of short vegetation (grassland and cropland) originally in each grid cell of the BECCS regions from the composite map. For the simulations, the vegetation type in these BECCS regions was changed into either the generic crop type (i.e., in the reference scenario) or a specific bioenergy crop type (i.e., in the bioenergy crop scenarios).

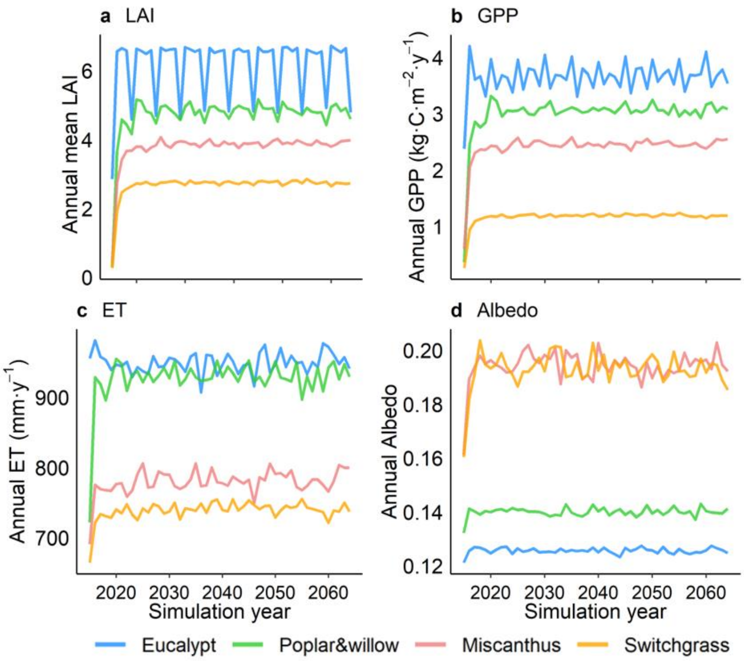

Supplementary Figure 12. Time series of LAI, GPP, ET, albedo during the 50-year simulations for the four bioenergy crops based on the idealized composite cultivation map.

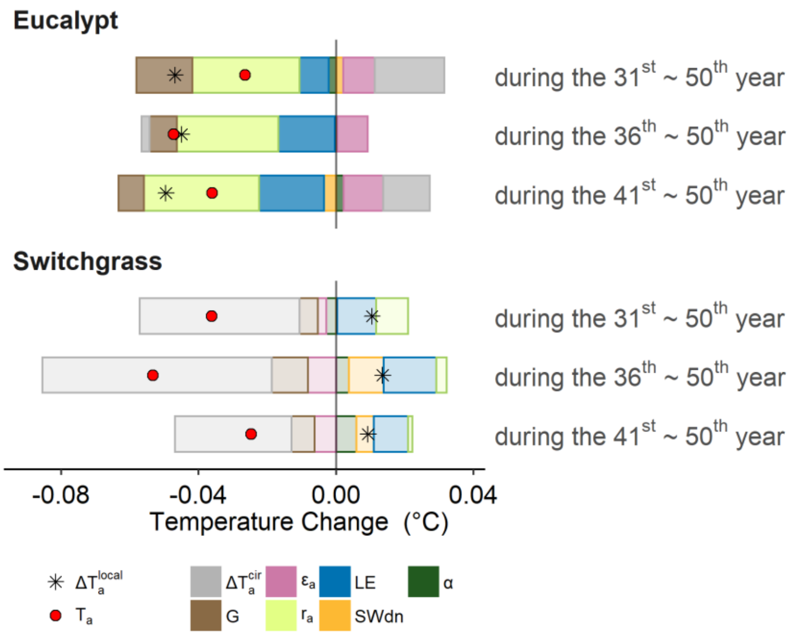

Supplementary Figure 13. Contributions of different components to air temperature changes at the global scale for the eucalypt and switchgrass cultivation using the composite cultivation map. Six rows of bars represent the results averaged over three different periods (i.e., the 41st – 50th year, the 36th – 50th year and the 31st – 50th year) of the simulations. Symbols are the same as in **Figure 2.**

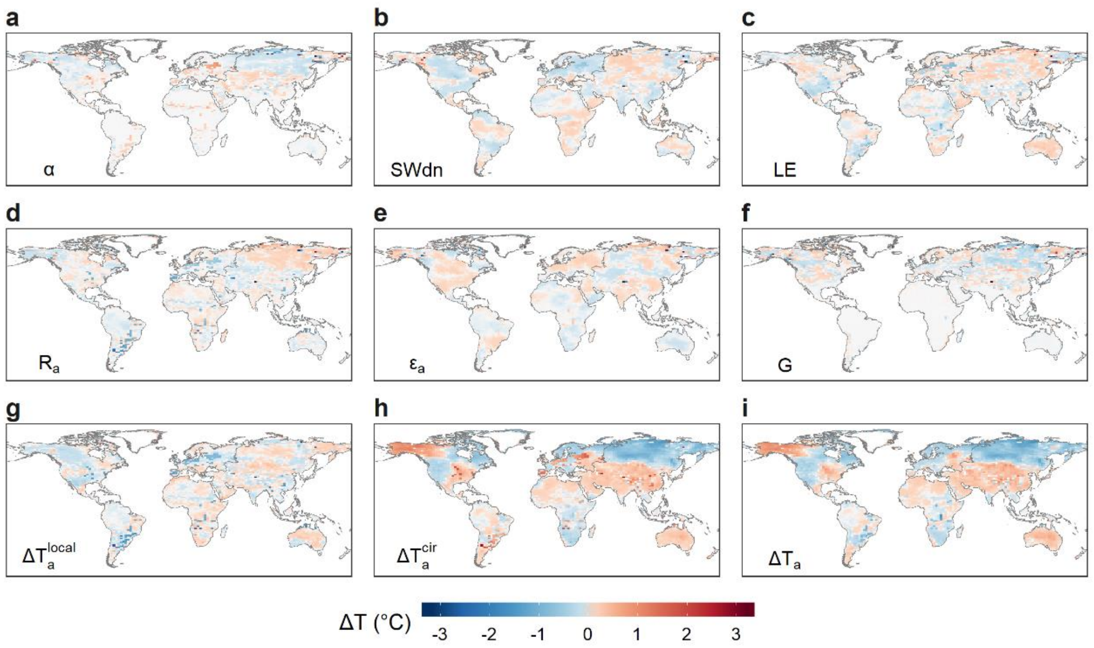

Supplementary Figure 14. Air temperature change in response to eucalypt cultivation using the composite cultivation map. **a-f** show the contribution of each component to air temperature change, with their combined effect shown in **g** (changes induced by altered local surface energy balance). **h** shows the air temperature change induced by atmospheric circulation. **i** shows the air temperature change (sum of **g** and **h**).

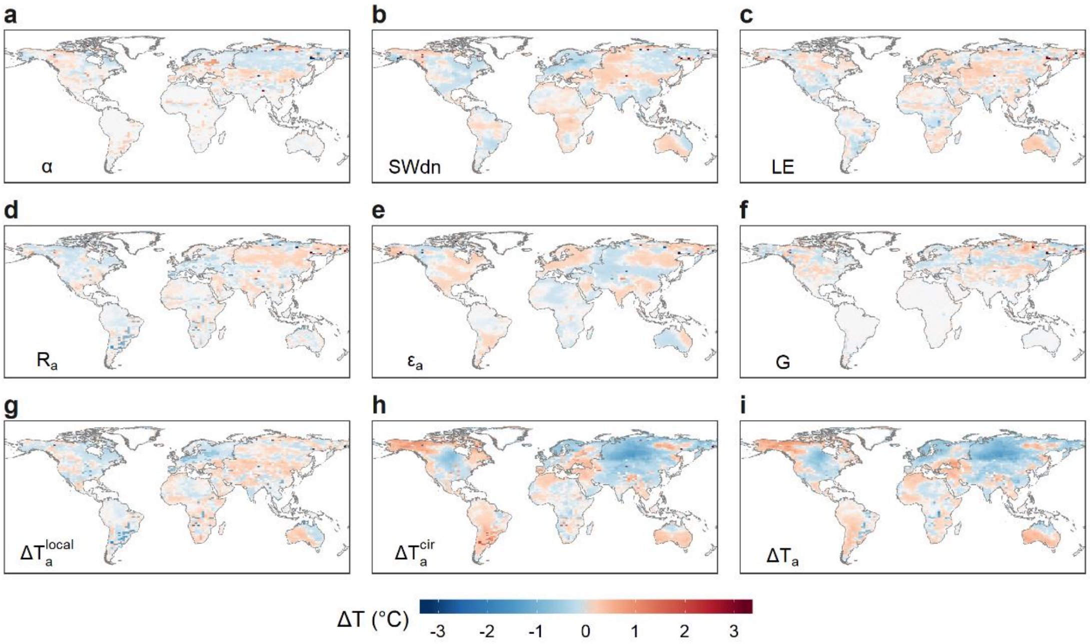

Supplementary Figure 15. Same as **Supplementary Figure 14** but for poplar & willow.

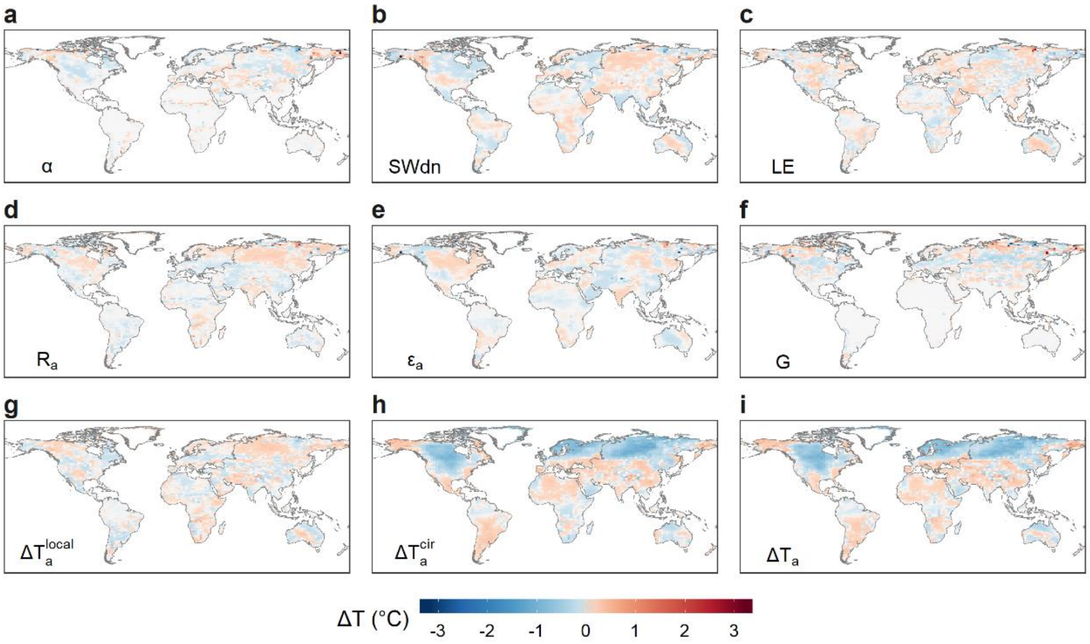

Supplementary Figure 16. Same as **Supplementary Figure 14** but for miscanthus.

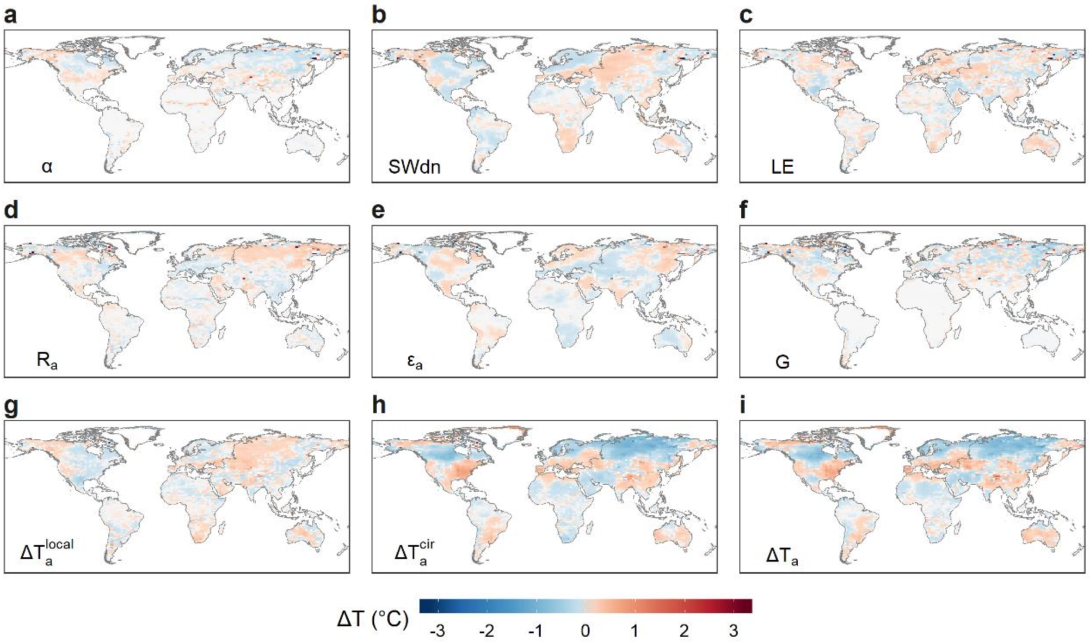

Supplementary Figure 17. Same as **Supplementary Figure 14** but for switchgrass.

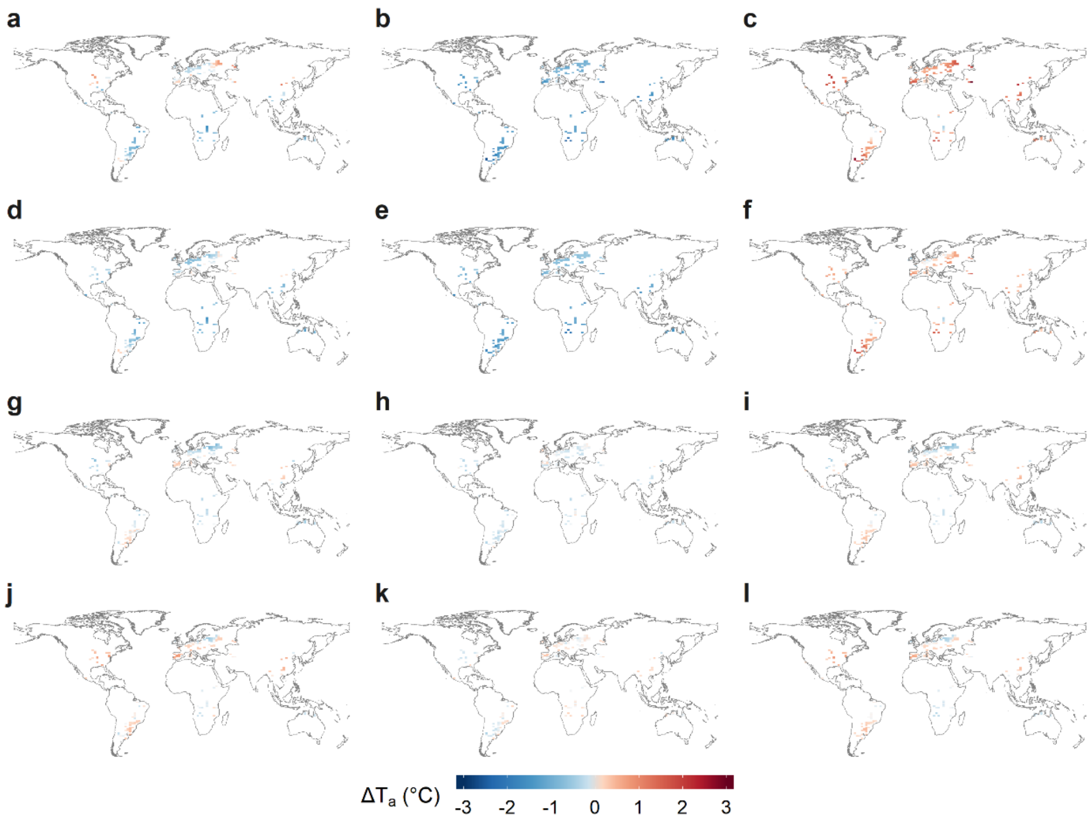

Supplementary Figure 18. Air temperature change in the BECCS regions based on the composite cultivation map. Each row represents air temperature change due to cultivation of a different bioenergy crop: eucalypt (**a-c**), poplar & willow (**d-f**), miscanthus (**g-i**) and switchgrass (**j-l**). The left hand column of panels show air temperature changes, while the middle and right hand columns show air temperature changes induced by altered local surface energy balance (ΔT_a^{local}) and atmospheric circulation changes (ΔT_a^{cir}), respectively.

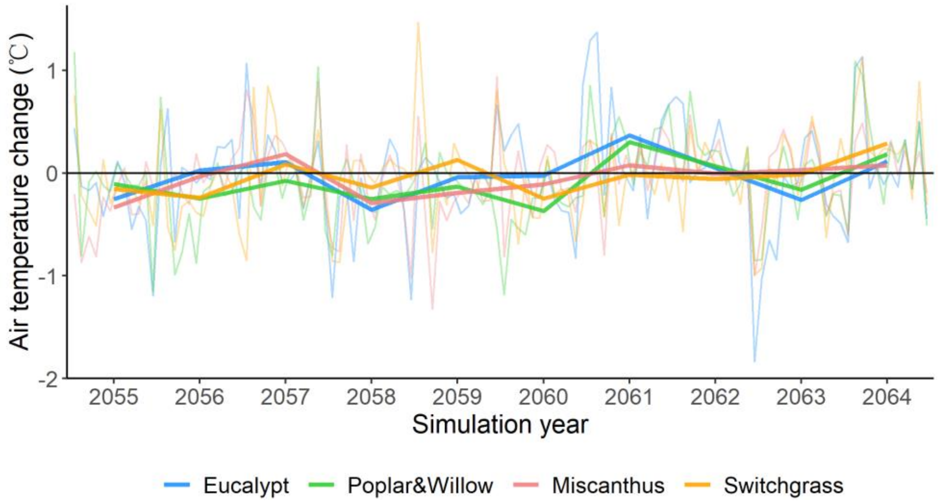

Supplementary Figure 19. Interannual variation of air temperature change in the four idealized bioenergy crop scenarios using the composite cultivation map during the final 10 years of the simulations. The bold lines indicate annual ΔT_a averaged over all grid cells, while the fine lines represent monthly ΔT_a.

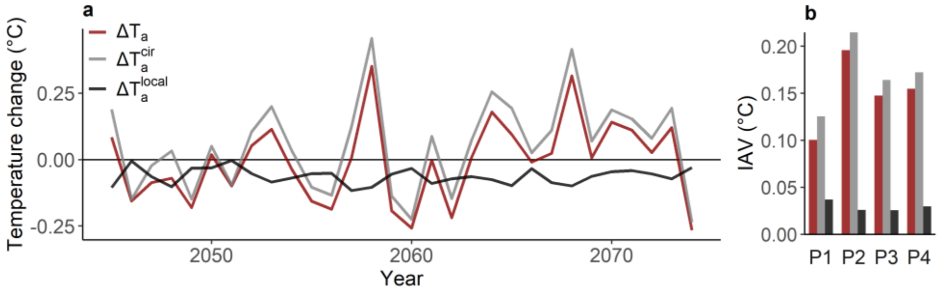

Supplementary Figure 20. Time series and interannual variability (IAV) of air temperature change in the eucalypt cultivation scenario based on the IMAGE map. **a** Time series of air temperature change (ΔT_a) and its two components induced by changes in local energy budget (ΔT_a^{local}) and atmospheric circulation (ΔT_a^{cir}) during the 31st to 60th year of the simulation. **b** IAV of ΔT_a, ΔT_a^{local} and ΔT_a^{cir} (same color as in a) over different periods of the simulation ("P1", "P2", "P3" and "P4" represent the periods of 31st – 40th year, the 41st – 50th year, the 51st – 60th year and the 31st – 60th year of the simulation).

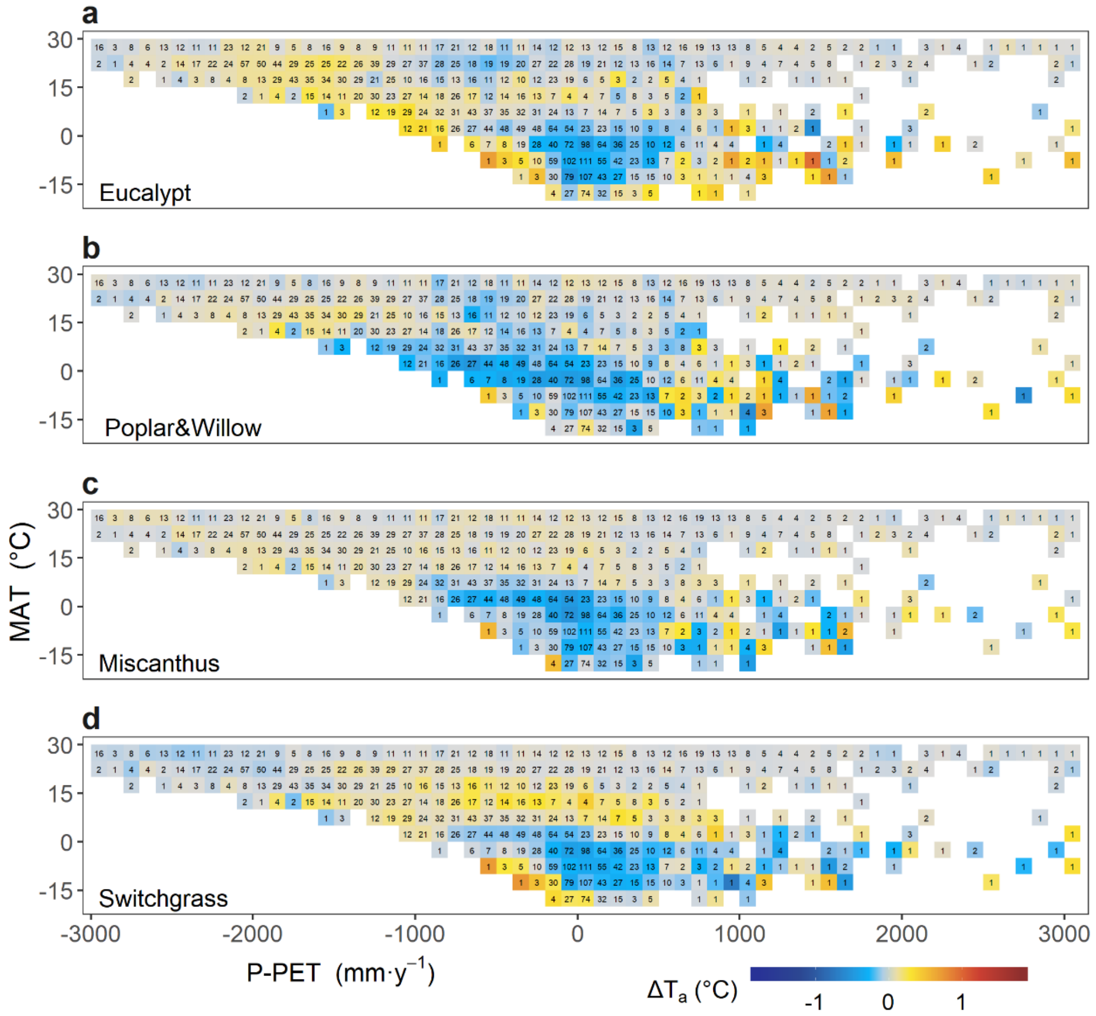

Supplementary Figure 21. Mean air temperature change in each MAT (mean annual temperature) and P-PET (difference between mean annual precipitation and mean annual potential evapotranspiration) interval. **a-d** represent T_a changes due to the cultivation of eucalypt (**a**), poplar & willow (**b**), miscanthus (**c**) and switchgrass (**d**) using the composite cultivation map.

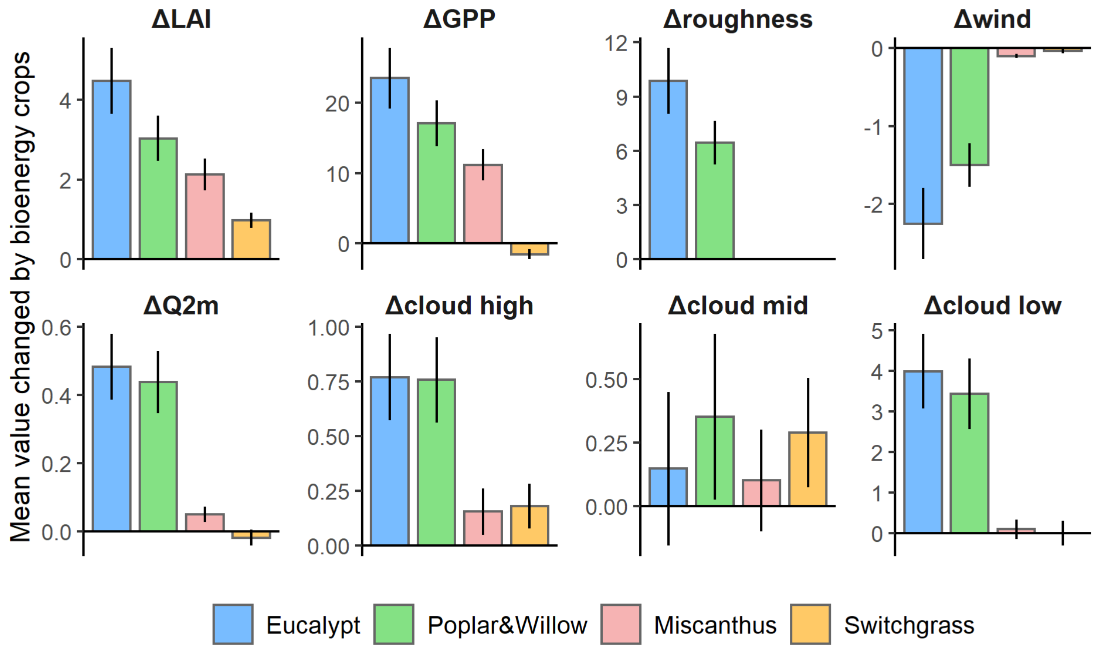

Supplementary Figure 22. Mean changes of various variables in response to bioenergy crop cultivation in the BECCS regions based on the composite cultivation map. The variables are LAI, GPP (kg m^{-2}), surface roughness height (“roughness”, m), surface wind speed at 10m height (m s^{-1}), specific air humidity at 2m height (“Q2m”, kg kg^{-1}), high cloud fraction (“cloud high”, %), medium cloud fraction (“cloud mid”, %), and low cloud fraction (“cloud low”, %). Error bars show the standard error of the changes.

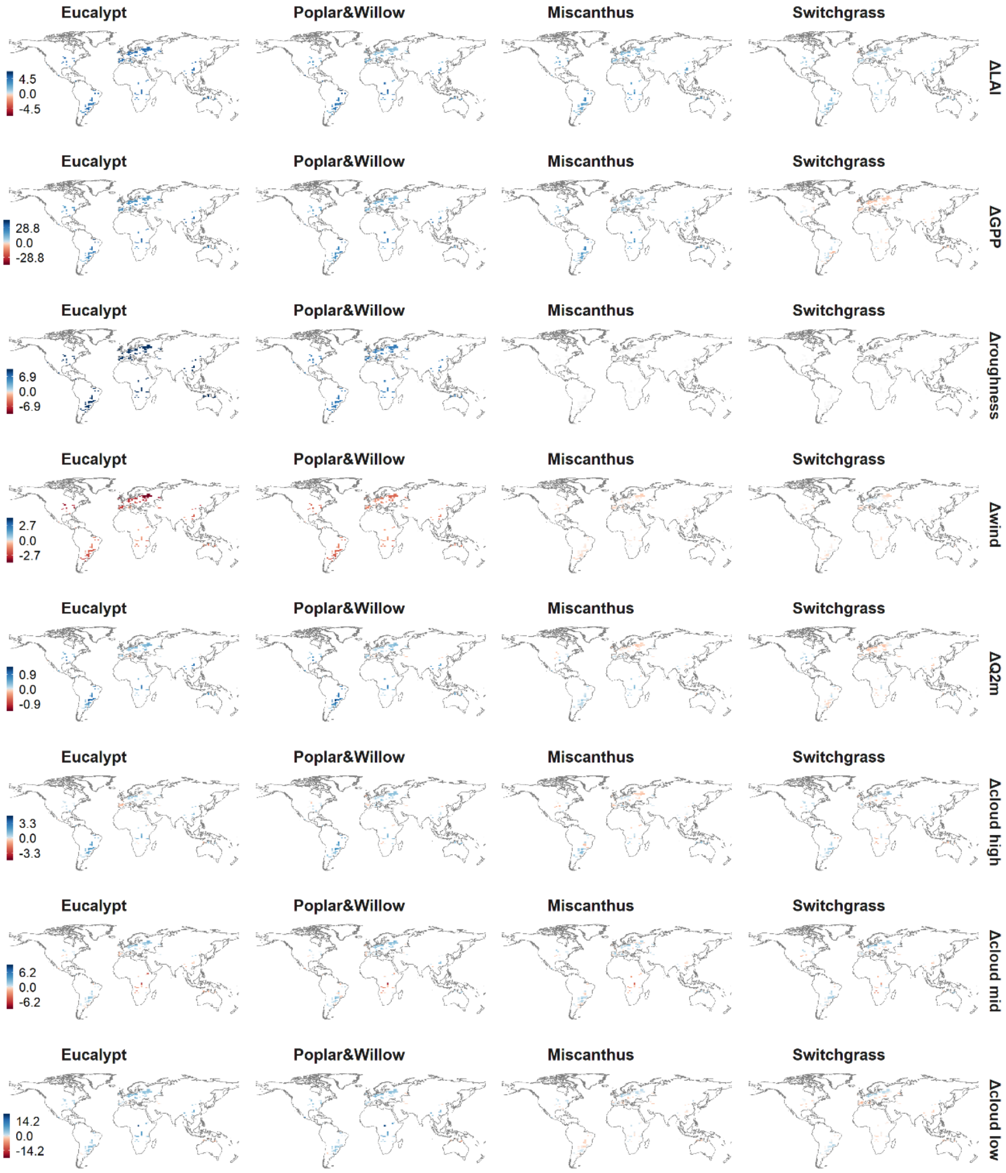

Supplementary Figure 23. Changes of relevant variables in response to bioenergy crop cultivation over the BECCS regions based on the composite cultivation map. The four columns represent variable changes due to cultivation of eucalypt, poplar & willow, miscanthus and switchgrass, respectively. The variables are LAI, GPP (kg m^{-2}), surface roughness height (“roughness”, m), surface wind speed at 10m height (m s^{-1}), specific air humidity at 2m height (“Q2m”, kg kg^{-1}), high cloud fraction (“cloud high”, %), medium cloud fraction (“cloud mid”, %), and low cloud fraction (“cloud low”, %).

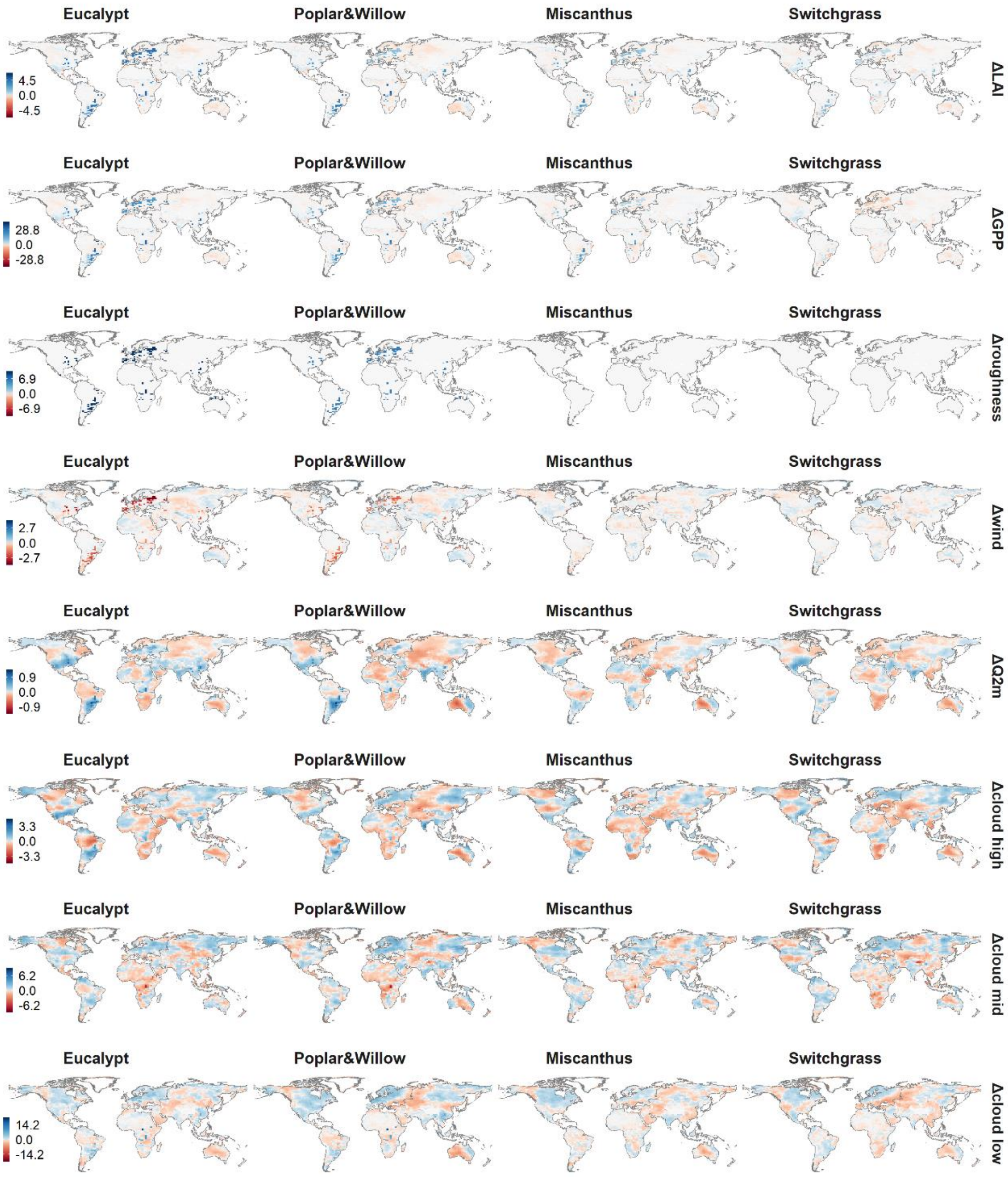

Supplementary Figure 24. Same as **Supplementary Figure 23** but for the entire global land surface.

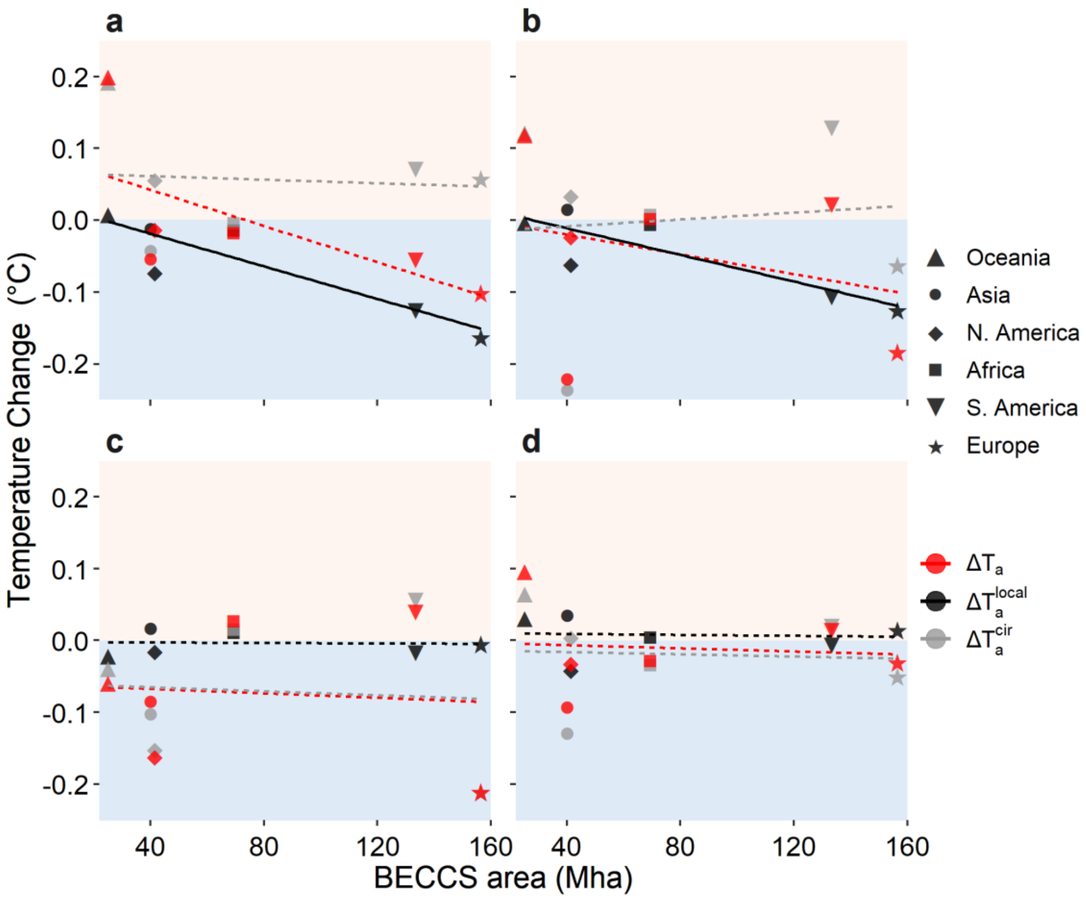

Supplementary Figure 25. BECCS area and temperature change based on the composite cultivation map (mean ΔT_a, ΔT_a^{local} and ΔT_a^{cir} over the whole continent) in different continents. **a-d** show temperature changes induced by cultivation of eucalypt (**a**), poplar & willow (**b**), miscanthus (**c**) and switchgrass (**d**). Background shading indicates the sign of the temperature change (red for **a** warming effect and blue for a cooling effect). The solid and dotted lines indicate significant correlations with $p<0.05$ and $p>0.05$ respectively.

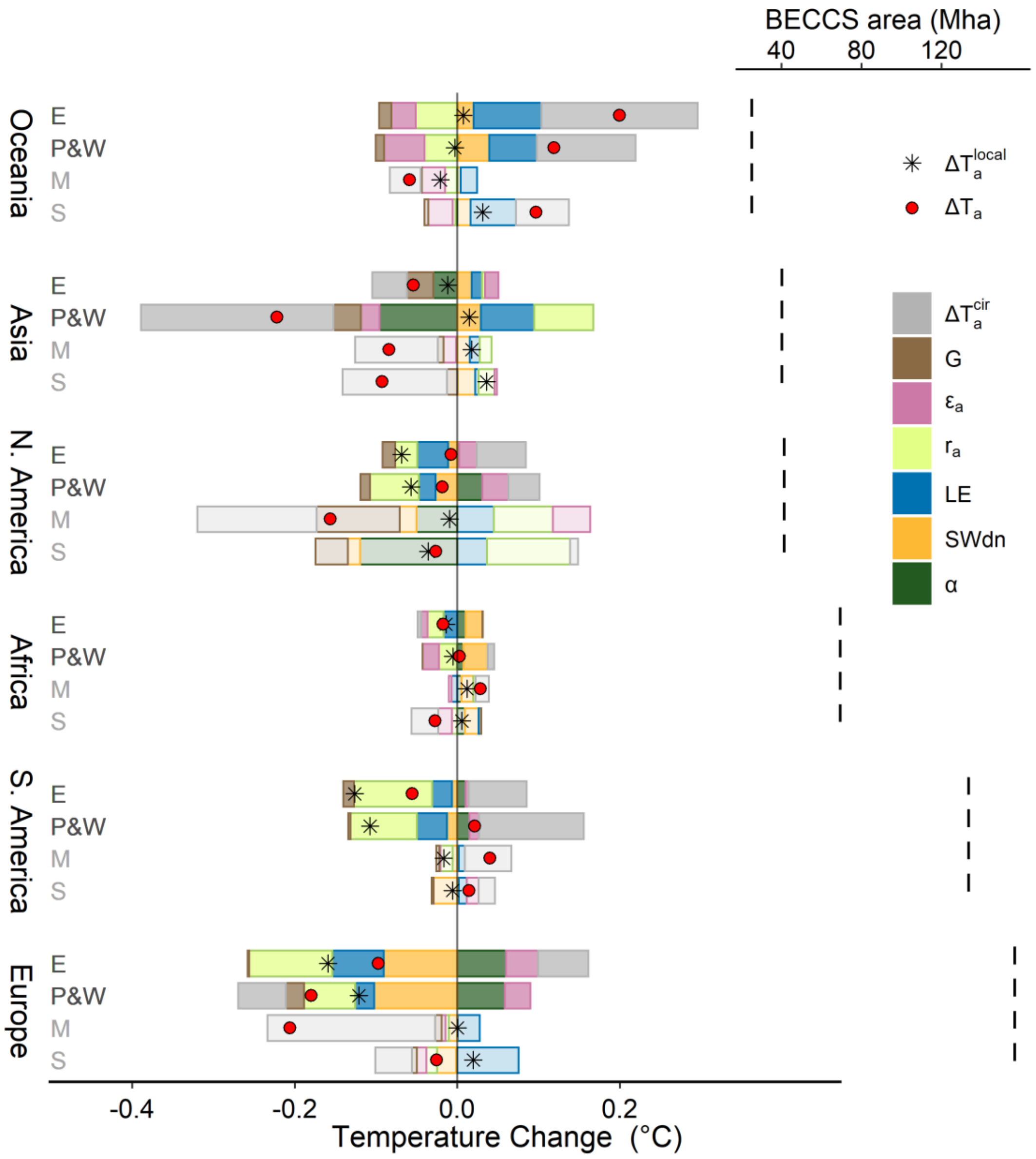

Supplementary Figure 26. Contributions of different components to air temperature changes in each continent for the four bioenergy crops (E: eucalypt; P&W: polar & willow; M: miscanthus; S: switchgrass) based on the composite cultivation map. Area of bioenergy cultivation (i.e., BECCS area) in each continent is shown on the right. Symbols in the bar plot are the same as in **Figure 2**.

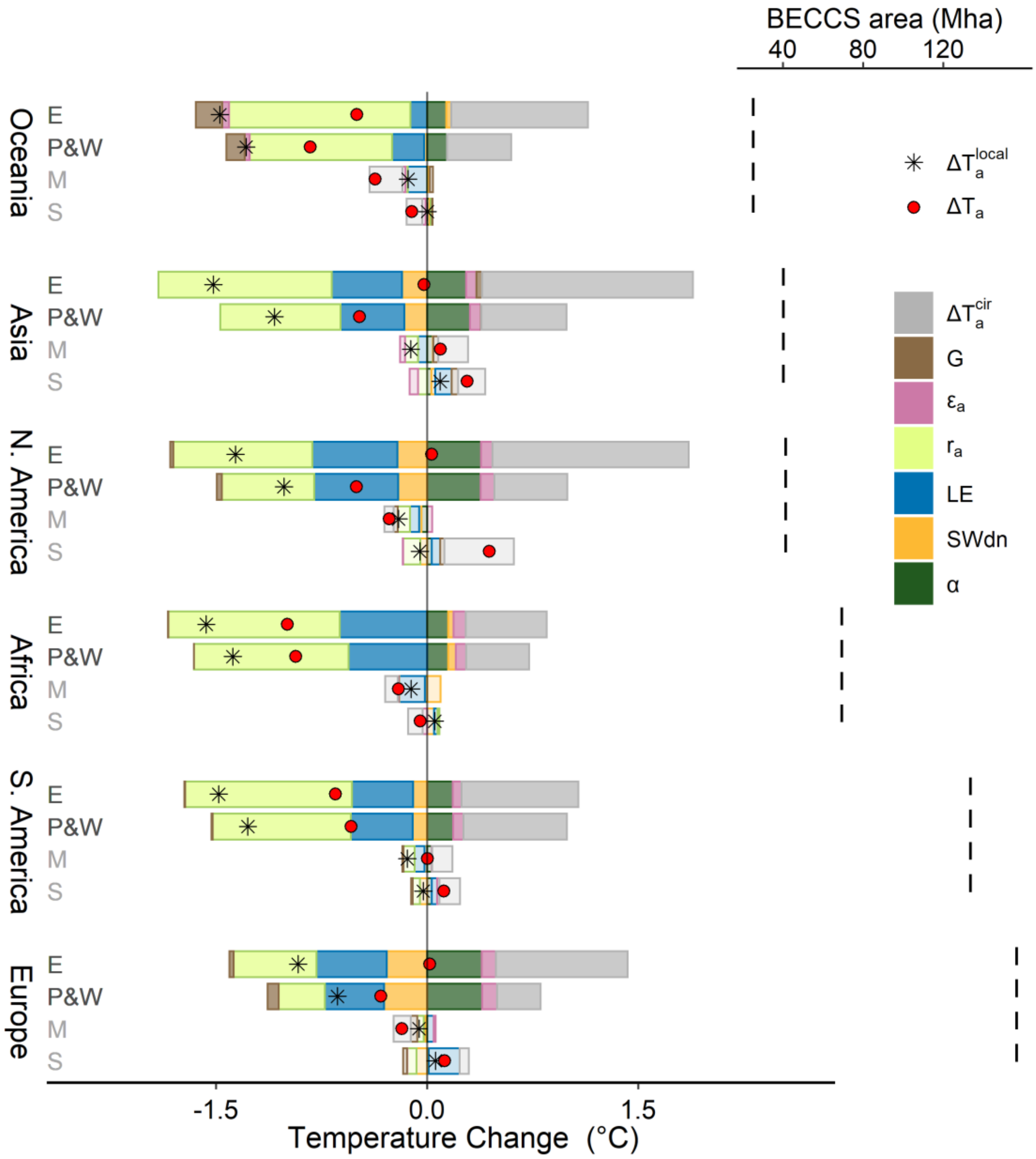

Supplementary Figure 27. Same as **Supplementary Figure 26** but for the BECCS regions.

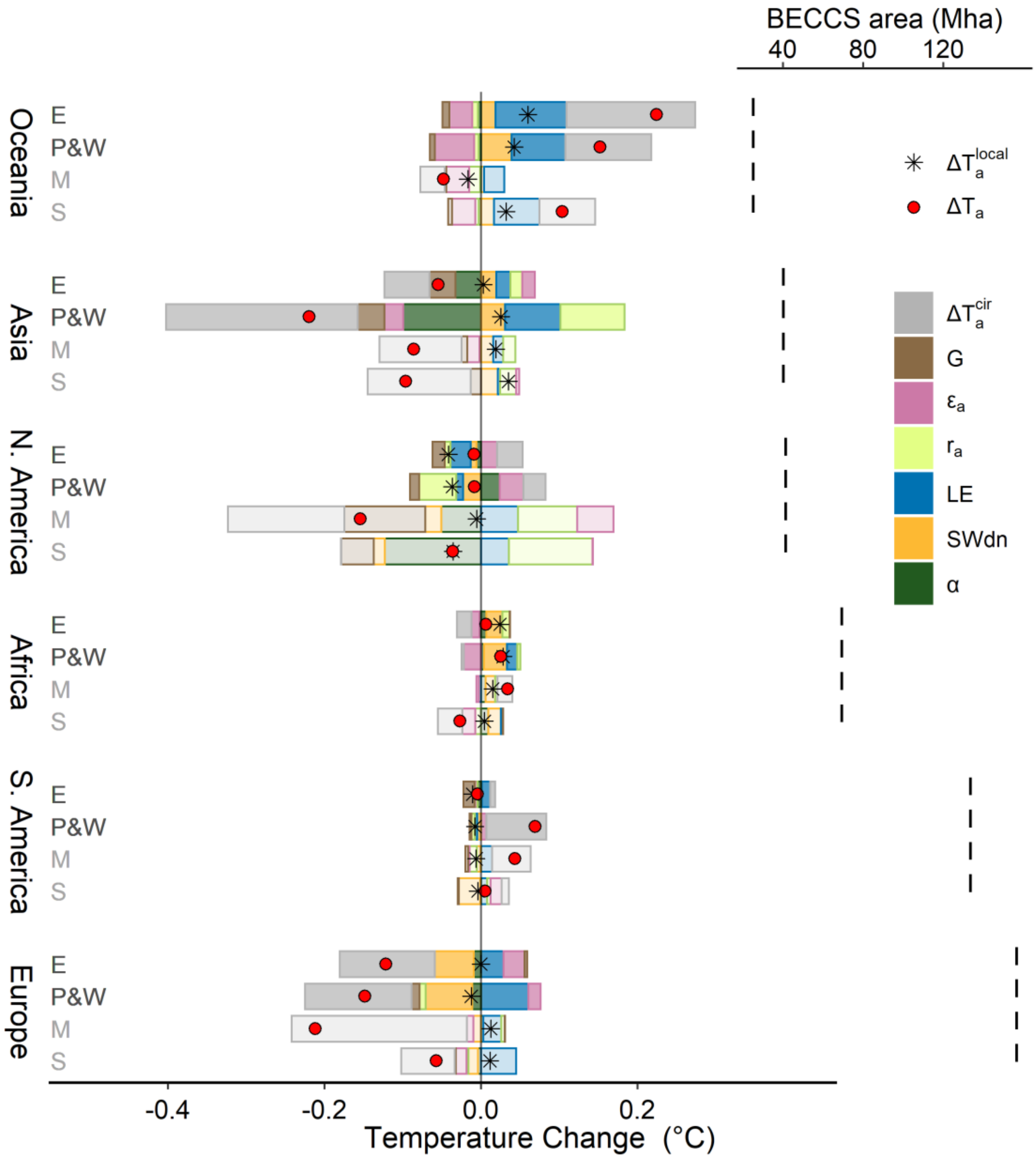

Supplementary Figure 28. Same as **Supplementary Figure 26** but outside the BECCS regions.

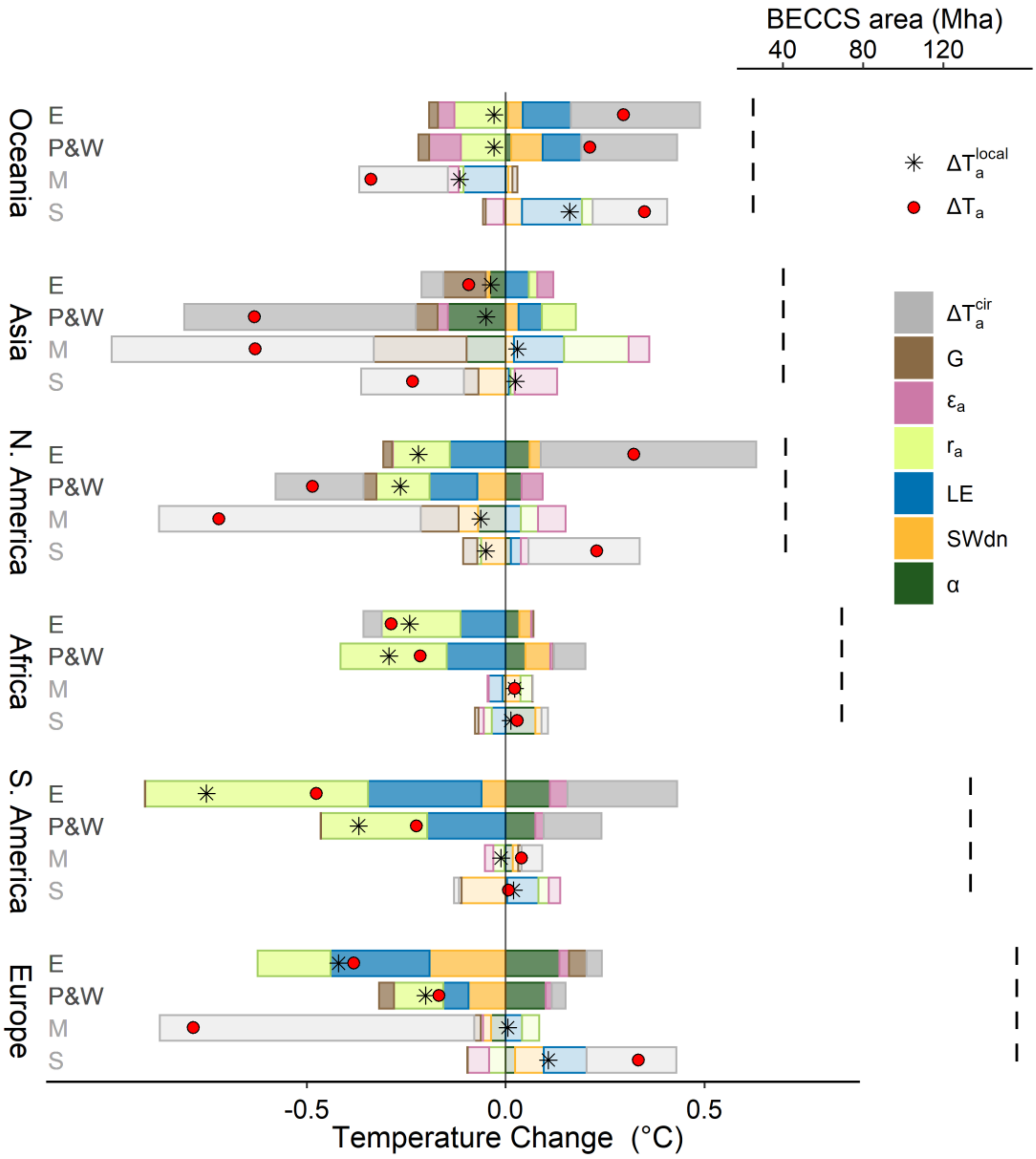

Supplementary Figure 29. Same as **Supplementary Figure 26** but for the grid cells with significant temperature changes in each scenario (i.e., grid cells with black shading in **Figure 1c-f**).

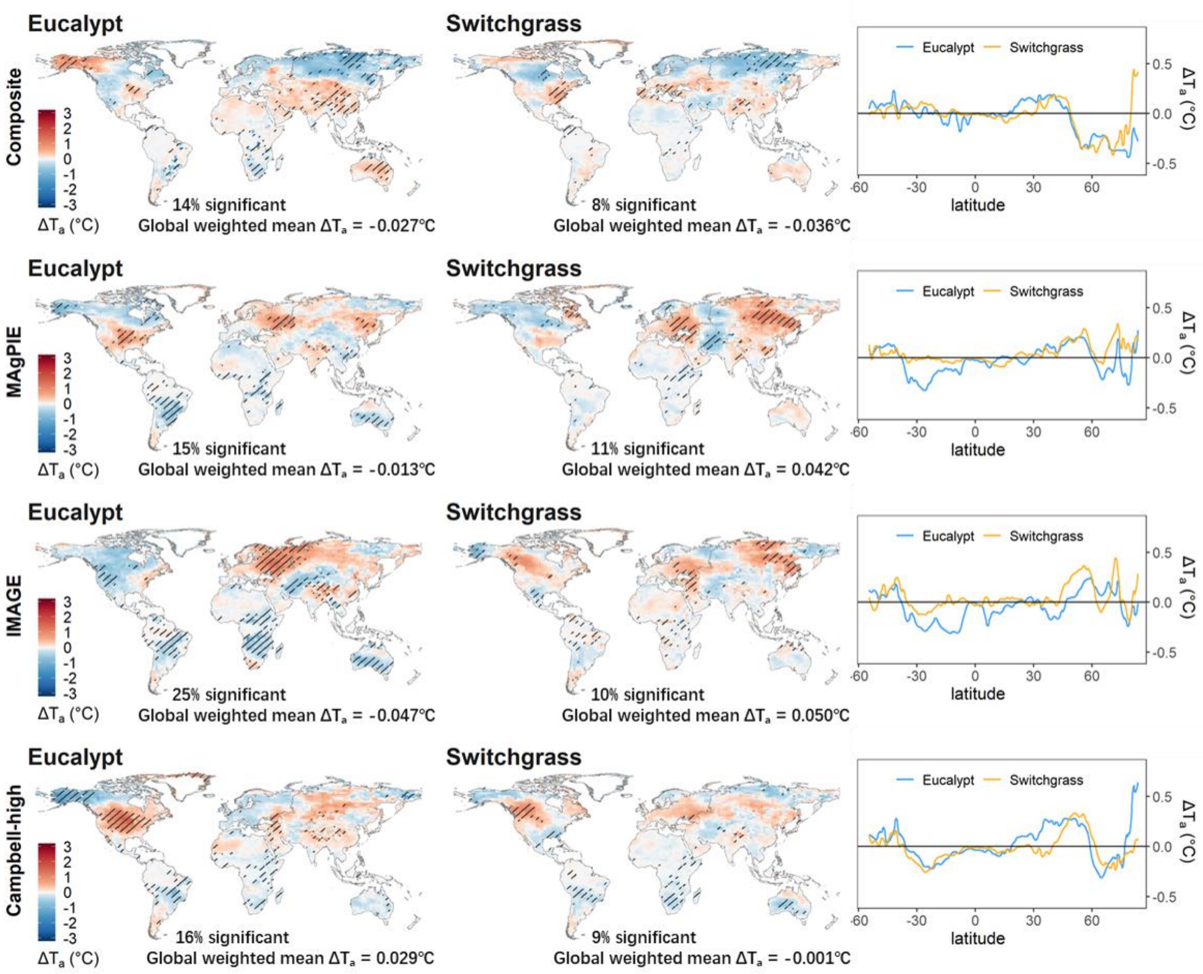

Supplementary Figure 30. Air temperature changes induced by cultivation of eucalypt and switchgrass based on various cultivation maps. Each row shows the results from one cultivation map (i.e., the composite map, and the individual maps from MAgPIE, IMAGE and Campbell-high). The left and middle columns are the spatial distribution of ΔT_a induced by cultivating eucalypt and switchgrass, and the zonal averages are shown in the right column. Black shading in the spatial distributions of ΔT_a indicates grid cells with a significant difference ($p<0.1$) in ΔT_a.

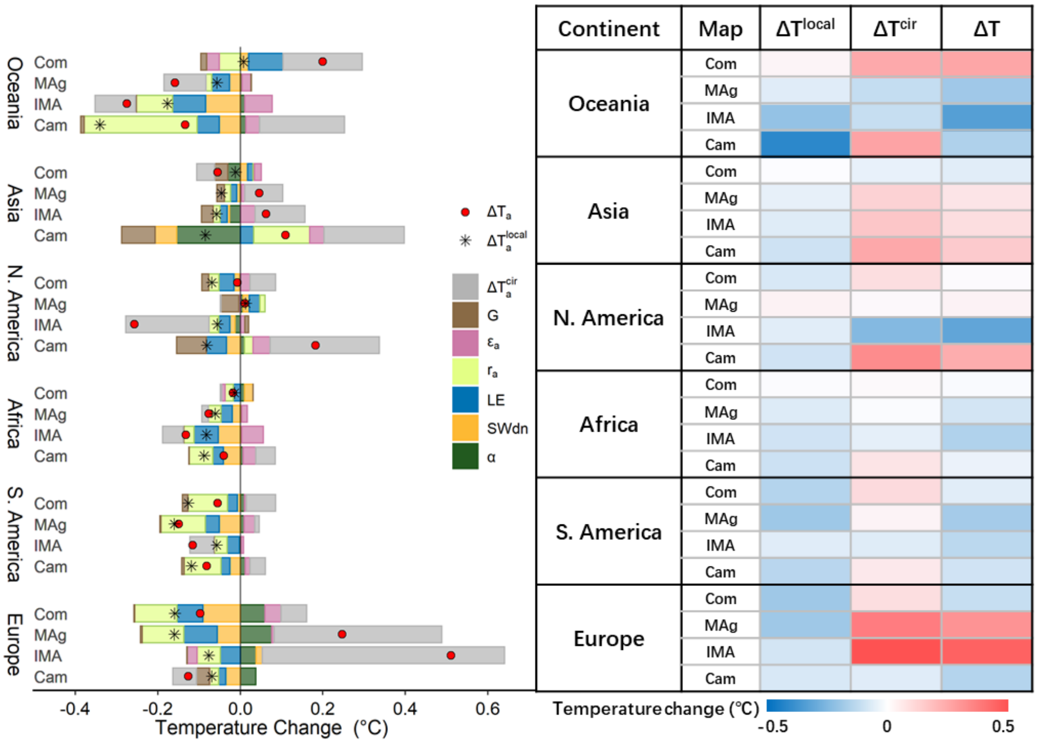

Supplementary Figure 31. Contributions of different components to air temperature changes at the continental scale for the eucalypt cultivation. Symbols in the bar plot are the same as in **Figure 2**. The table on the right shows temperature change (blue for cooling and red for warming) in each continent for different cultivation maps ("Com" for the composite map, "MAg" for MAgPIE, "IMA" for IMAGE, and "Cam" for Campbell-high).

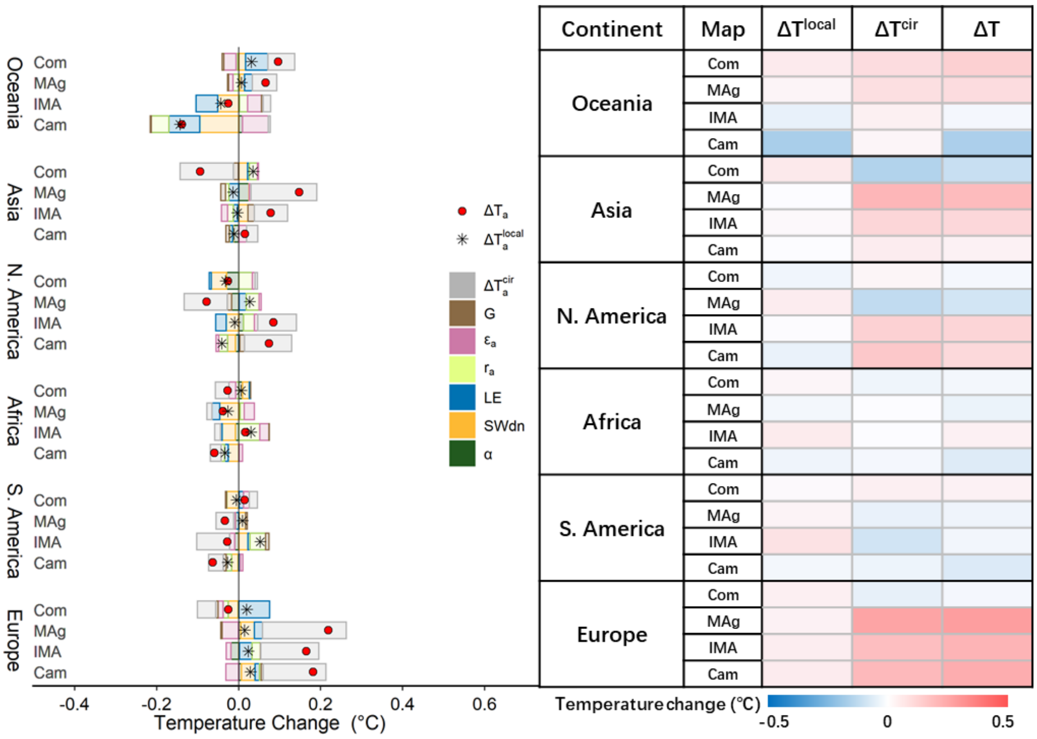

Supplementary Figure 32. Same as **Supplementary Figure 31**, but for the switchgrass cultivation.

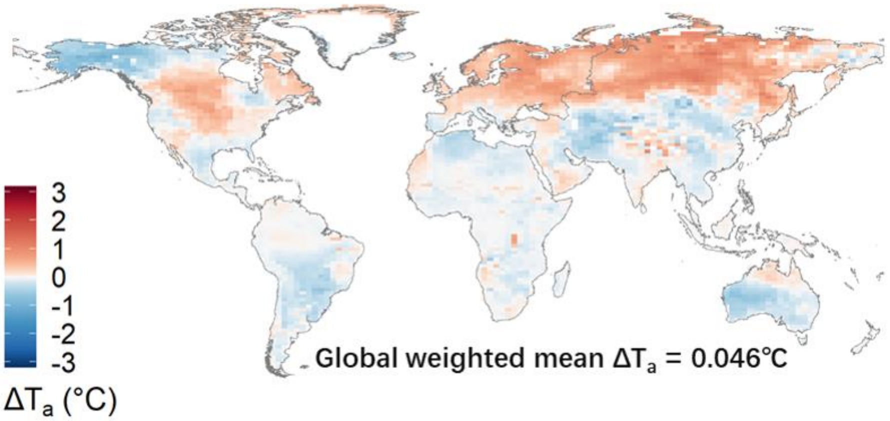

Supplementary Figure 33. Difference in air temperature between two reference simulations (S_{ref} - S_{ref}^{pre}).

References

1. Boucher, O. et al. Presentation and Evaluation of the IPSL‐CM6A‐LR Climate Model. *J. Adv. Model. Earth Sy.* **12**, (2020).
2. Krinner, G. et al. A dynamic global vegetation model for studies of the coupled atmosphere-biosphere system. *Global Biogeochem. Cy.* **19**, (2005).
3. Hourdin, F. et al. The LMDZ4 general circulation model: climate performance and sensitivity to parametrized physics with emphasis on tropical convection. *Clim. Dynam.* **27**, 787-813 (2006).
4. Contoux, C., Ramstein, G. & Jost, A. Modelling the mid-Pliocene Warm Period climate with the IPSL coupled model and its atmospheric component LMDZ5A. *Geosci. Model Dev.* **5**, 903-917 (2012).
5. Li, W. et al. ORCHIDEE-MICT-BIOENERGY: an attempt to represent the production of lignocellulosic crops for bioenergy in a global vegetation model. *Geosci. Model Dev.* **11**, 2249-2272 (2018).
6. Li, W., Ciais, P., Makowski, D. & Peng, S. A global yield dataset for major lignocellulosic bioenergy crops based on field measurements. *Scientific Data*. **5**, 180169 (2018).
7. Li, Y., Piao, S., Chen, A., Ciais, P. & Li, L. Z. X. Local and teleconnected temperature effects of afforestation and vegetation greening in China. *National Science Review*. **7**, 897-912 (2020).
8. Zeng, Z. et al. Climate mitigation from vegetation biophysical feedbacks during the past three decades. *Nat. Clim. Change*. **7**, 432-436 (2017).
9. Pastorello, G. et al. The FLUXNET2015 dataset and the ONEFlux processing pipeline for eddy covariance data. *Scientific Data*. **7**, (2020).
10. Cai, H. et al. Consideration of land use change-induced surface albedo effects in life-cycle analysis of biofuels. *Energ. Environ. Sci.* **9**, 2855-2867 (2016).
11. Luyssaert, S. et al. Trade-offs in using European forests to meet climate objectives. *Nature*. **562**, 259-262 (2018).
12. Georgescu, M., Lobell, D. B. & Field, C. B. Direct climate effects of perennial bioenergy crops in the United States. *P. Natl. Acad. Sci. Usa.* **108**, 4307-4312 (2011).
13. Robertson, G. P. et al. Cellulosic biofuel contributions to a sustainable energy future: Choices and outcomes. *Science*. **356**, (2017).
14. Kang, S. et al. Marginal Lands: Concept, Assessment and Management. *J. Agr. Sci.* **5**, 129-139 (2013).
15. Tang, Y., Xie, J. S. & Geng, S. Marginal Land-based Biomass Energy Production in China. *J. Integr. Plant Biol.* **052**, 112-121 (2010).
16. Campbell, J. E., Lobell, D. B., Genova, R. C. & Field, C. B. The Global Potential of Bioenergy on Abandoned Agriculture Lands. *Environ. Sci. Technol.* **42**, 5791-5794 (2008).
17. Cai, X., Zhang, X. & Wang, D. Land Availability for Biofuel Production. *Environmental Science & Technology: ES&T*. **45**, 334-339 (2011).
18. Popp, A. et al. Land-use transition for bioenergy and climate stabilization: model comparison of

drivers, impacts and interactions with other land use based mitigation options. *Climatic Change*. **123**, 495-509 (2014).

19. Stehfest, E. et al. Integrated assessment of global environmental change with IMAGE 3.0: Model description and policy applications. *Netherlands Environmental Assessment Agency (PBL)*, (2014).
20. Goldewijk, K. K. Estimating global land use change over the past 300 years: The HYDE Database. *Global Biogeochem. Cy.* **15**, 417-433 (2001).
21. Klein Goldewijk, K., Beusen, A., Van Drecht, G. & De Vos, M. The HYDE 3.1 spatially explicit database of human-induced global land-use change over the past 12,000 years. *Global Ecol. Biogeogr.* **20**, 73-86 (2011).
22. Peng, S. et al. Benchmarking the seasonal cycle of CO2 fluxes simulated by terrestrial ecosystem models. *Global Biogeochem. Cy.* **29**, 46-64 (2015).
23. Canadell, J. et al. Maximum rooting depth of vegetation types at the global scale. *Oecologia*. **108**, 583-595 (1996).
24. Kala, J. et al. Influence of Leaf Area Index Prescriptions on Simulations of Heat, Moisture, and Carbon Fluxes. *J. Hydrometeorol.* **15**, 489-503 (2014).
25. Ramstein, G., Serafini-Le Treut, Y., Le Treut, H., Forichon, M. & Joussaume, S. Cloud processes associated with past and future climate changes. *Clim. Dynam.* **14**, 233-247 (1998).
26. Bourgeois, Q. et al. Ubiquity and impact of thin mid-level clouds in the tropics. *Nat. Commun.* **7**, 12432 (2016).
27. Xu, X., Riley, W. J., Koven, C. D., Jia, G. & Zhang, X. Earlier leaf-out warms air in the north. *Nat. Clim. Change*. **10**, 370-375 (2020).
28. Graham, E. A., Mulkey, S. S., Kitajima, K., Phillips, N. G. & Wright, S. J. Cloud cover limits net CO2 uptake and growth of a rainforest tree during tropical rainy seasons. *P. Natl. Acad. Sci. Usa.* **100**, 572-576 (2003).
29. Chen, T., Rossow, W. B. & Zhang, Y. Radiative Effects of Cloud-Type Variations. *J. Climate*. **13**, 264-286 (2000).
30. MacDougall, A. H. The Transient Response to Cumulative CO2 Emissions: a Review. *Current Climate Change Reports*. **2**, 39-47 (2016).
31. Tokarska, K. B. et al. Recommended temperature metrics for carbon budget estimates, model evaluation and climate policy. *Nat. Geosci.* **12**, 964-971 (2019).
32. Gillett, N. P., Arora, V. K., Matthews, D. & Allen, M. R. Constraining the Ratio of Global Warming to Cumulative CO2 Emissions Using CMIP5 Simulations*. *J. Climate*. **26**, 6844-6858 (2013).
33. Matthews, H. D., Gillett, N. P., Stott, P. A. & Zickfeld, K. The proportionality of global warming to cumulative carbon emissions. *Nature*. **459**, 829-832 (2009).
